# conMItion: an R package adjusting confounding factors for associations in multi-omics

**DOI:** 10.1093/bioinformatics/btag472

**Published:** 2026-06-30

**Authors:** Gaojianyong Wang, Frank Liu, Ze Chen, Teresa Davoli

**Affiliations:** Institute for Systems Genetics and Department of Biochemistry and Molecular Pharmacology, NYU Grossman School of Medicine, New York, NY 10016, United States; Present Address: Department of Bioinformatics, Max Planck Institute for Molecular Genetics, Berlin 14195, Germany; Institute for Systems Genetics and Department of Biochemistry and Molecular Pharmacology, NYU Grossman School of Medicine, New York, NY 10016, United States; Institute for Systems Genetics and Department of Biochemistry and Molecular Pharmacology, NYU Grossman School of Medicine, New York, NY 10016, United States; Institute for Systems Genetics and Department of Biochemistry and Molecular Pharmacology, NYU Grossman School of Medicine, New York, NY 10016, United States

## Abstract

**Summary:**

Association measurements, such as mutual information (MI), are fundamental in the analysis of cancer multi-omics data for identifying cancer-related genes, gene signatures, and gene regulatory networks, thereby shedding light on tumor development, progression, and treatment. Confounding factors, including tumor purity and mutation burden, can bias association measurements in MI, potentially leading to the misclassification of passenger events as drivers. Conditional mutual information (CMI) provides a robust framework for assessing both linear and nonlinear associations while effectively accounting for different confounding factors. An R package called conMItion is introduced to estimate CMI and its statistical significance for multi-omics data, with the flexibility to adjust for one or two confounding factors. We demonstrated the utilization of conMItion through two use cases. First, we identified interchromosomal somatic copy number alteration–expression associations in bladder cancer. Second, we identified associated cell types within the lung cancer tumor microenvironment using single-cell RNA sequencing datasets.

**Availability and implementation:**

The conMItion package is freely available on CRAN at https://CRAN.R-project.org/package=conMItion. The two use cases described in the paper can be accessed at https://github.com/GJYWang/conMItion.

## 1 Introduction

Association measurement is one of the most fundamental tools for multi-omics analysis in cancer research, revealing the complex interactions driving tumor development and progression. For example, copy-number-dependent oncogenes and tumor suppressor genes have been identified through associations between somatic copy number alterations (SCNAs) and gene expression (Akavia *et al*. 2010). Co-expressed gene signatures have been identified as biomarkers of cancer hallmarks (Carter *et al*. 2006). In addition, association measurements can reveal gene regulatory networks (Aibar *et al*. 2017), providing insights into new drug targets.

Mutual information (MI) is a robust association measurement method for quantifying both linear and nonlinear dependencies in cancer (Daub *et al.* 2004). However, confounding factors, e.g. tumor purity (Carter *et al*. 2012) and mutation burden (Niknafs *et al*. 2023), can bias association analyses (Aran *et al*. 2015). Without proper adjustment for these confounders, association analyses may mistakenly identify passenger events associated with tumor purity or mutation burden as tumor drivers.

Therefore, addressing these confounding factors can improve the accuracy and reliability of biological inferences from association measurements. Conditional mutual information (CMI) is an ideal framework (Cover and Thomas 2006), as it evaluates both linear and nonlinear associations while theoretically accounting for an unlimited number of confounding factors. We have developed an R package called conMItion to estimate CMI for biological data. In this paper, we present two illustrative use cases of conMItion. The first identifies associated interchromosomal SCNA–expression pairs while controlling for tumor purity and mutation burden as confounding variables. The second evaluates cell-type abundance associations within the tumor microenvironment (TME), while controlling for tumor purity.

## 2 Methods

### 2.1 MI and CMI estimation using B-spline functions

MI quantifies the information shared between two random variables *X* and *Y* and is expressed as:


I(X;Y)=H(X)−H(X|Y)=H(X)+H(Y)−H(X,Y)

**Figure 1:**
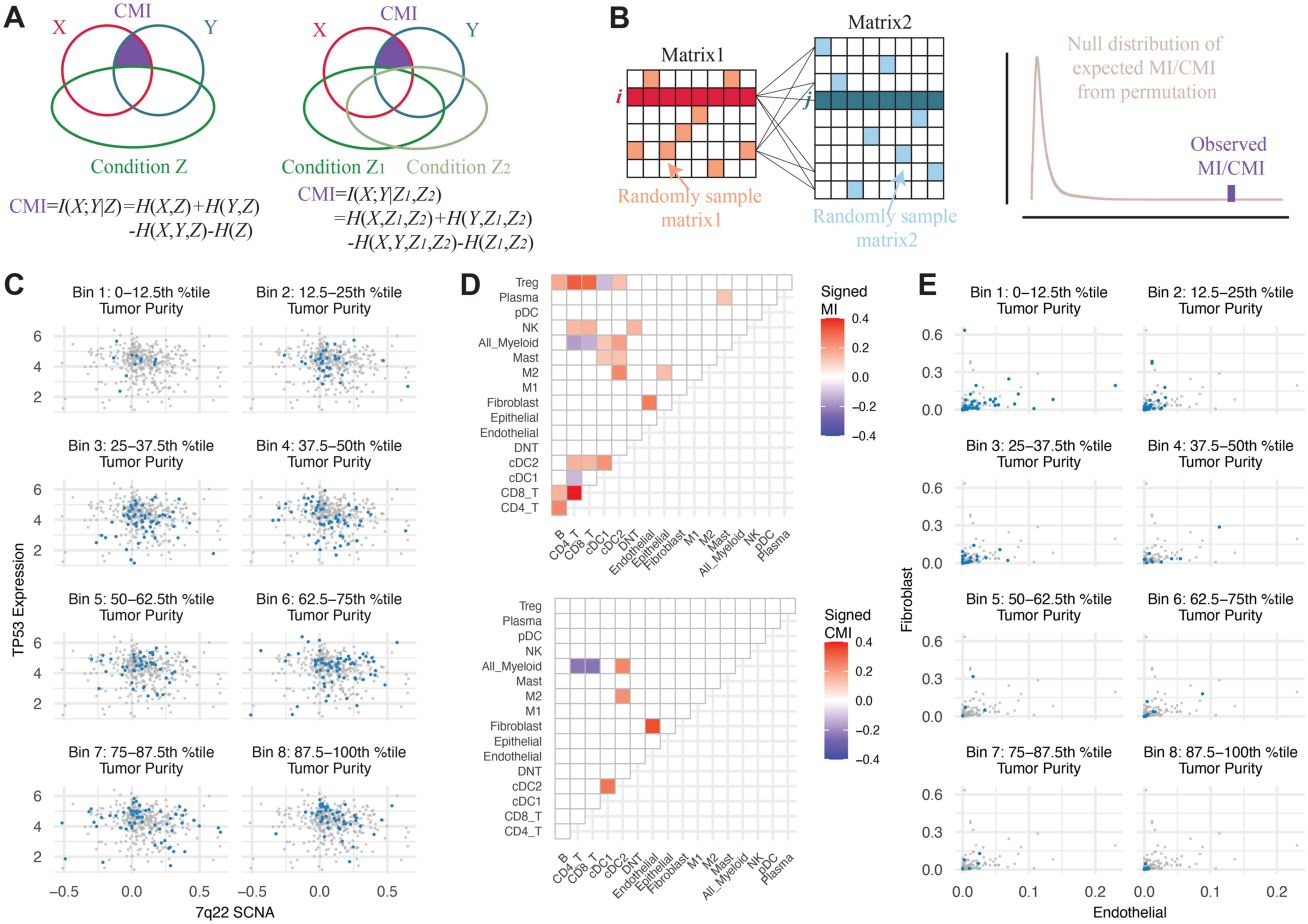
Mathematical fundamentals, workflow, and results of the conMItion package. (A) Venn diagram illustrating CMI between variables *X* and *Y* given the condition variable *Z* (left panel) and given condition variables $Z_{1}$ and $Z_{2}$ (right panel). The red and blue circles respectively represent the entropy H(X) and H(Y). The intersection of the red and blue circles represents MI I(X;Y). The shaded area (purple) represents CMI I(X;Y|Z) (left panel) and $I(X;Y|Z_{1},Z_{2})$ (right panel). (B) Illustration of conMItion estimating statistical significance through permutation. (C) Scatter plots of the SCNA of 7q22, containing *KMT2E*, and the expression of *TP53* in BLCA with tumor purity and mutation burden as confounding factors. Tumor purity is split into eight bins. Mutation burden is not shown. (D) Statistically significant (FDR<0.2) associations between cell fractions within lung adenocarcinoma (MI: upper panel, CMI: lower panel) with tumor purity as the confounding factor. The sign was given by Spearman correlation. (E) Scatter plots of endothelial and fibroblast cell fractions within lung adenocarcinoma with tumor purity as the confounding factor. Tumor purity is split into eight bins.

Where H(·) denotes Shannon entropy (Supplementary Document, available as supplementary data at *Bioinformatics* online). CMI quantifies the information shared between *X* and *Y* given a third condition variable *Z* (Fig. 1A) and is defined as:


I(X;Y∣Z)=H(X,Z)+H(Y,Z)−H(X,Y,Z)−H(Z)

CMI evaluates the dependency between *X* and *Y* while excluding the influence of *Z*. It can be extended to account for two conditions (Fig. 1A, Supplementary Document, available as supplementary data at *Bioinformatics* online).

Marginal and joint probabilities must be estimated before calculating the CMI in multi-omics data. For a variable *X* with observed values $x_{1},\ldots ,x_{N}$, these values are normalized between 0 and 1: $\hat{x}=(x-x_{\text{min}})/(x_{\text{max}}-x_{\text{min}})$.

The range between 0 and 1 is divided into *B* bins of the same size, represented by $X_{1},\ldots ,X_{B}$. B-spline functions (Daub *et al.* 2004) are used to estimate the probability mass function of p(X) (Supplementary Document, available as supplementary data at *Bioinformatics* online). A knot vector $t_{i}$ is defined by a number of bins i=1,…,B and a spline order of integer s,0<s<B:


$$t_{i}:=\{\begin{array}{cc} 0, & \text{if}\;i<s\\ \frac{i-s+1}{B-s+1}, & \text{if}\;s\leq i\leq B-1\\ 1, & \text{if}\;i>B-1 \end{array}$$

B-spline basis functions $B_{i,s}(\hat{x})$ are recursively constructed (Supplementary Document) as follows:


$$B_{i,1}(\hat{x})=\{\begin{array}{cc} 1, & \text{if}\;t_{i}\leq \hat{x}<t_{i+1}\\ 0, & \text{otherwise} \end{array}$$

$$B_{i,s}(\hat{x})=\frac{\hat{x}-t_{i}}{t_{i+s-1}-t_{i}}B_{i,s-1}(\hat{x})+\frac{t_{i+s}-\hat{x}}{t_{i+s}-t_{i+1}}B_{i+1,s-1}(\hat{x})$$

The probability mass function is then estimated as $p(X_{i})=\sum_{v=1}^{N}B_{i,s}({\hat{x}}_{v})/N$. Joint probabilities for two and three variables are respectively given as $p(X_{i},Y_{j})=\sum_{v=1}^{N}B_{i,s}({\hat{x}}_{v})\times B_{j,s}({\hat{y}}_{v})/N$ and $p(X_{i},Y_{j},Z_{k})=\sum_{v=1}^{N}B_{i,s}({\hat{x}}_{v})\times B_{j,s}({\hat{y}}_{v})\times B_{k,s}({\hat{z}}_{v})/N$. These estimated marginal and joint probability mass functions can be used to compute CMI (and MI) according to their standard definitions (Supplementary Document, available as supplementary data at *Bioinformatics* online). The obtained MI or CMI is normalized by max(I(X;X),I(Y;Y)) to have a maximum value of 1 and a minimum value of 0, facilitating comparisons across datasets.

### 2.2 conMItion package

conMItion has been developed for association measurements in multi-omics data and assessing their statistical significance. Compared with correlation, partial correlation, and linear regression, MI and CMI can capture nonlinear dependencies, with CMI additionally enabling adjustment for nonlinear confounding effects (Supplementary Document, available as supplementary data at *Bioinformatics* online). The current implementation supports MI and CMI for one or two condition variables. Core MI and CMI estimations are implemented in compiled C to improve computational efficiency.

The package accommodates different input data formats, such as a matrix and a vector (Supplementary Document, available as supplementary data at *Bioinformatics* online) or two matrices. In the matrix–vector format, MI or CMI is calculated between each row of the matrix and a numeric vector. In the matrix–matrix format, MI or CMI is calculated between each row of the first matrix and each row of the second matrix.

Users may specify the bin number and spline order for MI and CMI estimation. Based on our sensitivity analysis (Supplementary Document, available as supplementary data at *Bioinformatics* online), we recommend that the bin number not exceed one tenth of the sample size and the spline order be one quarter to one third of the bin number. When these parameters are not supplied, conMItion automatically selects these parameters based on the sample size.

### 2.3 Statistical significance assessment

To assess whether the observed CMI is statistically significant, conMItion adopts a permutation-based randomization procedure (Fig. 1B). Consider the case of two matrices as input (for matrix-vector input, see Supplementary Document, available as supplementary data at *Bioinformatics* online), with their rows representing features such as expressions, SCNAs, mutations, or cell fractions and their columns representing samples. The null hypothesis is that the two input features (one from each matrix) are not associated with one another, given the variables being conditioned on.

conMItion generates a null distribution of *D* CMI values. In each of *D* iterations, one vector is generated independently per input matrix by randomly selecting one entry from each sample column and then randomly permuting the resulting vector (Fig. 1B). CMI is calculated between the two randomly constructed vectors (while the variable(s) being conditioned on preserve their original order). This procedure disrupts associations between the two matrices, generating a null distribution of CMI expected from randomized vectors. The empirical *P*value is defined as the fraction of this null distribution greater than or equal to the observed CMI (Fig. 1B).

conMItion also supports a conditional permutation scheme to preserve the dependence between the randomly sampled vector(s) and the condition variable. The condition variable is first divided into bins, and one sampled vector is permuted only within each bin rather than across all samples. This permutation process preserves the dependency between the sampled vector(s) and the condition variable while disrupting the association between them (Supplementary Document, available as supplementary data at *Bioinformatics* online).

Generation of null distributions can be distributed to independent jobs, with each job starts from the same seed but skips the random permutations assigned to earlier batches before generating its own permutation statistics. This ensures that different jobs produce non-overlapping portions of the overall permutation sequence. A null distribution of 100 million values was generated from matrices containing approximately 400 samples with 30 000 and 1500 features in about 1.5 hours using 500 jobs distributed across 256 nodes (Section 3.1, Supplementary Document, available as supplementary data at *Bioinformatics* online).

## 3 Results

### 3.1 Interchromosomal SCNA-expression association

conMItion was applied to Urothelial Bladder Carcinoma (BLCA) samples to identify interchromosomal SCNA-expression associations. We collected expression data for 29 353 genes, and obtained average SCNA levels for 1368 genomic segments that together comprise the whole genome (Supplementary Methods, available as supplementary data at *Bioinformatics* online). Across 393 patient samples, conMItion estimated the CMI between the expression of these genes and the SCNAs of these genomic segments (approximately 40 million pairs) while conditioning on tumor purity and mutation burden (termed CMI${\;}_{p,m}$), using a bin number of eight and a spline order of two (Supplementary Document, available as supplementary data at *Bioinformatics* online). Furthermore, conMItion calculated MI without conditioning and CMI conditioned only on tumor purity (CMI${\;}_{p}$) using the same parameters.

We found that *TP53* expression demonstrated an association with SCNAs of 7q22.1–q22.3 (102.7–105.2 Mb, Fig. 1C, ${\text{CMI}}_{p,m}=0.15$, $P=1.1\times 10^{-6}$, $\mathrm{FDR}=2.8\times 10^{-4}$; MI=0.04, $P=8.1\times 10^{-4}$, $\mathrm{FDR}=7.9\times 10^{-3}$), which contains *KMT2E*. Although our association analysis does not establish a causal relationship, this observation is biologically notable because downregulation of *KMT2E* has previously been implicated in p53 activation in the DNA damage response (Cheng *et al*. 2011). The association between *LCN12* expression and SCNA at 2q34–q35 (209.7–214.7 Mb) was stronger when overall mutation burden was not controlled (MI=0.10, $P<10^{-8}$, $\mathrm{FDR}<1.5\times 10^{-6}$; ${\text{CMI}}_{p}=0.10$, $P=2.6\times 10^{-4}$, FDR=0.012). However, ${\text{CMI}}_{p,m}$ for this expression-SCNA pair was 0.065 (P=0.18, FDR=0.26), suggesting the magnitude of the association inflated by mutation burden as a confounding variable. Similarly, the association between *ITGAX* expression and SCNA at 9q22.31–22.32 (92.5–94.3 Mb) decreased in magnitude (MI=0.13, $P<10^{-8}$, $\mathrm{FDR}<1.5\times 10^{-6}$) after conditioning on tumor purity (MI=0.051, P=0.23, FDR=0.31), suggesting that the observed MI for this expression-SCNA pair could be inflated by tumor purity. These examples demonstrate how conMItion can distinguish between genuine interchromosomal SCNA-expression associations of interest and spurious associations driven by confounding factors, improving the accuracy of identifying intricate interactions in cancer.

### 3.2 Cell type associations in the TME

Twelve single-cell RNA sequencing (scRNA-seq) datasets of lung cancer were collected and processed. Cell type annotation was performed through the detection of canonical marker genes, and the fractional abundance of every cell type within the TME was computed across all samples (Supplementary Methods, available as supplementary data at *Bioinformatics* online).

Signed MI and CMI (conditioned on tumor purity) were calculated between all cell type fractions using a bin number of eight and a spline order of two. The association sign was set as the sign of the corresponding Spearman correlation (Fig. 1D). Co-occurring cell type pairs included CD$4^{+}$ T cells with CD$8^{+}$ T cells (CMI=0.20, P=0.011, FDR=0.22), and endothelial cells (EC) with fibroblasts (FIB) (CMI=0.33, $P=1.3\times 10^{-6}$, $\mathrm{FDR}=1.8\times 10^{-4}$, Fig. 1E). Additionally, associations between the Treg fraction and various lymphocytes (B cells, CD$4^{+}$ T cells, and CD$8^{+}$ T cells) that were found statistically significant by MI (0.17, 0.31, 0.29, respectively; FDR=0.011, $<10^{-5}$, $<10^{-5}$, respectively) became insignificant after conditioning on tumor purity (CMI=0.11, 0.14, 0.15, respectively; FDR=1.0,1.0,1.0, respectively), suggesting the magnitude of these associations may be confounded by tumor purity. This use case illustrates how conMItion’s ability to condition on confounding factors like tumor purity can both highlight previously obscured associations (endothelial-fibroblast) and filter out spurious correlations (Treg-lymphocyte), providing a more nuanced understanding of cell type co-localization patterns in the TME.

## 4 Discussion and conclusion

This paper presents the R package conMItion, which uses CMI to control for the effects of confounding factors in association measurements in multi-omics analyses. Through two use cases, we demonstrated that conMItion offers additional insights into the complex interchromosomal SCNA-expression patterns and cell type co-localization patterns in cancer.

In the first use case, we identified an association between *TP53* expression and SCNAs containing *KMT2E* in BLCA after adjusting for tumor purity and mutation burden (Cheng *et al*. 2011). This illustrates a common challenge in cancer genomics: linking copy number to gene expression under nonlinear confounding. Although tools such as ABSOLUTE (Carter *et al*. 2012) can adjust SCNA estimates for tumor purity and ploidy, an analogous correction is less straightforward for bulk RNA-seq. Because measured expression reflects a mixture of malignant and nonmalignant cells and cannot be reliably rescaled to a pure tumor value, studying the relationship between copy number and expression remains challenging (Cheng *et al*. 2022). A common workaround is to adjust for tumor purity using partial correlation or linear regression, but these approaches remove only linear purity effects. If purity is nonlinearly related to SCNA or expression, residual confounding may remain and distort the apparent association. By conditioning on tumor purity through CMI, conMItion accounts for nonlinear dependencies between purity and SCNA or expression while capturing nonlinear SCNA–expression associations, without assuming a global linear relationship. This enables DNA–RNA association analysis while accounting for nonlinear confounding factors.

In the second use case, we found that the positive association between FIBs and ECs (Frangogiannis 2020) in the TME becomes more apparent after accounting for tumor purity. This finding is biologically meaningful, as cancer-associated FIBs are known to promote angiogenesis and support EC function through the secretion of pro-angiogenic factors such as *VEGF*, *FGF2*, and chemokines (Sahai *et al*. 2020). Moreover, ECs can undergo endothelial-to-mesenchymal transition (EndMT) to become a source of cancer-associated FIBs in the TME, providing a direct mechanistic link between these two cell types (Zeisberg *et al*. 2007). The stronger CMI compared to MI indicates that this stromal interaction occurs consistently across varying levels of tumor purity, suggesting it represents a fundamental feature of tumor-stroma crosstalk rather than an artifact of tumor cell content.

Importantly, our analysis also revealed that associations between Tregs and other lymphocyte populations (B cells, CD$4^{+}$ cells, and CD$8^{+}$ T cells) that appeared significant by MI became non-significant when calculating CMI, indicating that these associations were driven by tumor purity rather than specific cellular interactions. This observation is biologically plausible, as Tregs are a specialized immunosuppressive subset of CD$4^{+}$ T cells characterized by *FOXP3* expression, functioning to suppress antitumor immunity rather than directly collaborating with conventional effector lymphocytes. While Tregs normally comprise only 4% of CD$4^{+}$ cells in peripheral blood, they can constitute 20–30% of CD$4^{+}$ cells in the TME. Their accumulation in tumors occurs through preferential chemotaxis (e.g. via CCR4-CCL22 gradients) and reflects their role in creating an immunosuppressive microenvironment enabling tumor immune evasion (Sakaguchi *et al*. 2020). Thus, the apparent associations between Tregs and other lymphocytes detected by MI likely reflect co-enrichment patterns related to overall immune infiltration (inversely correlated with tumor purity) rather than functional co-localization or direct cellular interactions.

Together, these examples demonstrate how conMItion can distinguish between genuine biological associations of interest versus spurious correlations driven by confounding variables, and underscore the importance of proper confounder adjustment in multi-omics association analyses.

## Supplementary Material

btag472_Supplementary_Data

## Data Availability

The data underlying this article are available in the GitHub repository at https://github.com/GJYWang/conMItion.
