# Supplementary material for: conMItion: an R package adjusting confounding factors for associations in multi-omics
Source: Bioinformatics. 2026 Jun 30;42(7):btag472. doi: 10.1093/bioinformatics/btag472 (PMC13378457; doi:10.1093/bioinformatics/btag472)
Supplement: btag472_Supplementary_Data [file btag472_supplementary_data.pdf]

conMItion: an R package adjusting confounding factors for associations in multi-omics
Supplementary Material

Contents

1 Statistical framework **2**
1.1 Information theory basics . . . 2
1.2 CMI estimation using B-spline functions . . . 2
1.3 Statistical significance assessment for matrix-vector data format . . . 4

2 Data pre-processing **5**
2.1 Bladder cancer data . . . 5
2.2 Single cell RNA-sequencing dataset of lung cancer . . . 5

3 Parameter settings and sensitivity analysis **6**
3.1 Sensitivity analysis and guidance for selecting bin number and spline order for CMI . . . 6
3.2 Impact of bin number and spline order to identifying co-occurring mutation and CNV . . . 8

4 Comparison between MI/CMI and correlation/linear regression **10**
4.1 Definitions . . . 10
4.2 Comparing MI vs Pearson/Spearman correlation . . . 10
4.3 Comparing CMI vs partial correlation . . . 11
4.4 Comparing MI/CMI vs (partial) correlation in lung cancer data . . . 13
4.5 Comparing MI vs Spearman correlation in lung cancer data . . . 16

5 Runtime analysis **20**

6 Associated mutation-SCNA pairs in BLCA **22**

7 Evaluation of permutation schemes under simulated conditional-null **28**

1 Statistical framework

1.1 Information theory basics

Entropy quantifies the amount of information needed to describe a random variable. For a discrete random variable X with probability mass function $p(x)$ over possible outcomes $\mathcal{X}$, the entropy is defined as:

$$H(X) = -\sum_{x\in\mathcal{X}} p(x)\log_2 p(x)$$

The base-2 logarithm ensures that entropy is measured in bits. The joint entropy quantifies the amount of information required to describe two or more random variables. For two discrete random variables X and Y with joint probability mass function $p(x, y)$ over all possible outcomes $\mathcal{X} \times \mathcal{Y}$, the joint entropy is defined as:

$$H(X, Y) = -\sum_{x,y\in\mathcal{X}\times\mathcal{Y}} p(x, y)\log_2 p(x, y)$$

For three variables X, Y, and Z, with joint probability mass function $p(x, y, z)$ over all possible outcomes $\mathcal{X} \times \mathcal{Y} \times \mathcal{Z}$, the joint entropy is defined as:

$$H(X, Y, Z) = -\sum_{x,y,z\in\mathcal{X}\times\mathcal{Y}\times\mathcal{Z}} p(x, y, z)\log_2 p(x, y, z)$$

Mutual information (MI), denoted $I(X; Y)$, quantifies the information shared between two random variables X and Y. It represents the reduction in uncertainty of X given Y and is expressed as:

$$\begin{aligned} I(X; Y) &= H(X) - H(X|Y) \\ &= H(X) + H(Y) - H(X, Y) \end{aligned}$$

Conditional mutual information (CMI), denoted $I(X; Y|Z)$, quantifies the information shared between X and Y given a third condition variable Z. It is defined in terms of entropy as:

$$\begin{aligned} I(X; Y \mid Z) &= H(X \mid Z) - H(X \mid Y, Z) \\ &= H(X, Z) + H(Y, Z) - H(X, Y, Z) - H(Z) \end{aligned}$$

CMI evaluates the dependency between X and Y while excluding the influence of Z. It can be extended to account for multiple conditions. For condition variables Z_1 and Z_2, the CMI, $I(X; Y|Z_1, Z_2)$, is defined in terms of entropy as:

$$\begin{aligned} I(X; Y \mid Z_1, Z_2) &= H(X \mid Z_1, Z_2) - H(X \mid Y, Z_1, Z_2) \\ &= H(X, Z_1, Z_2) + H(Y, Z_1, Z_2) \\ &\quad - H(X, Y, Z_1, Z_2) - H(Z_1, Z_2) \end{aligned}$$

1.2 CMI estimation using B-spline functions

Marginal and joint probabilities must be estimated before calculating the CMI in continuous data, such as the multi-omics data. For a variable X with observed values $x_1, \ldots, x_N$, these values are normalized between 0 and 1:

$$\hat{x} = \frac{x - x_{\min}}{x_{\max} - x_{\min}}$$

The range between 0 and 1 is divided into B bins of the same size, represented by $\mathcal{X}_1, ..., \mathcal{X}_B$. B-spline functions [1] are used to estimate the probability mass function of $p(\mathcal{X})$. A knot vector t_i (Fig. 1) is defined based upon the bin number i and spline order s where $i, s \in \mathbb{Z}$ and $1 \le i, s \le B$:

$$t_i := \begin{cases} 0, & \text{if } i < s \\ \frac{i-s+1}{B-s+1}, & \text{if } s \le i \le B-1 \\ 1, & \text{if } i > B-1 \end{cases}$$

Intuitively, the knot vector splits the range from 0 to 1 into $B - s + 1$ evenly spaced segments (Fig. 1).

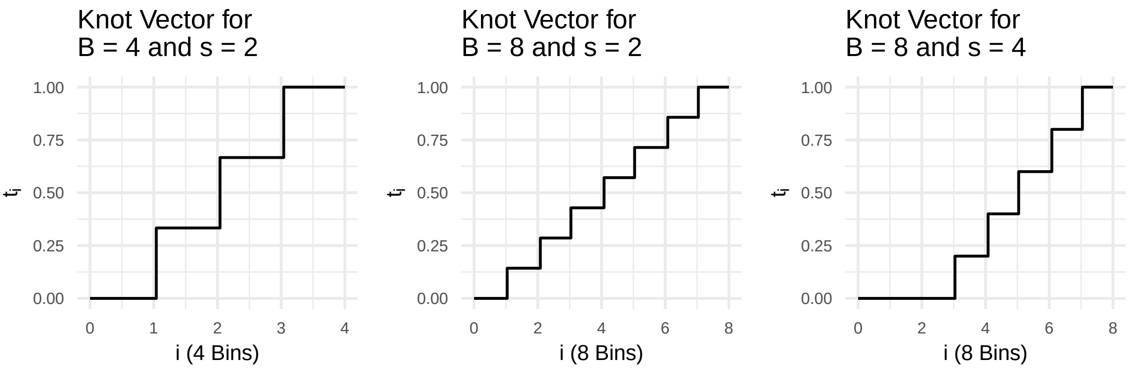

Figure 1: Illustration of knot vectors with varying choices for bin number and spline order.

B-spline basis functions $B_{i,s}(\hat{x})$ are constructed in order to map $\hat{x}$ data values into appropriate bins. B-spline basis functions $B_{i,s}(\hat{x})$ are recursively constructed as follows:

$$B_{i,1}(\hat{x}) = \begin{cases} 1, & \text{if } t_i \le \hat{x} < t_{i+1} \\ 0, & \text{otherwise} \end{cases}$$

$$B_{i,s}(\hat{x}) = \frac{\hat{x} - t_i}{t_{i+s-1} - t_i} B_{i,s-1}(\hat{x}) + \frac{t_{i+s} - \hat{x}}{t_{i+s} - t_{i+1}} B_{i+1,s-1}(\hat{x}) \qquad (\ s > 1)$$

B-spline basis functions with $s > 1$ can assign each data point $\hat{x}$ into multiple bins (Fig. 2), thus providing an estimation of association that is more robust to impact from outliers [1]. The probability mass function is then estimated as:

$$p(\mathcal{X}_i) = \frac{1}{N} \sum_{v=1}^{N} B_{i,s}(\hat{x}_v)$$

where $B_{i,s}(\hat{x}_v)$ represents the probability of $\hat{x}_v$ belonging to bin i given spline order s. The sum of such B-spline functions over all N observed data points is used to estimate the probability of bin i, $p(\mathcal{X}_i)$.

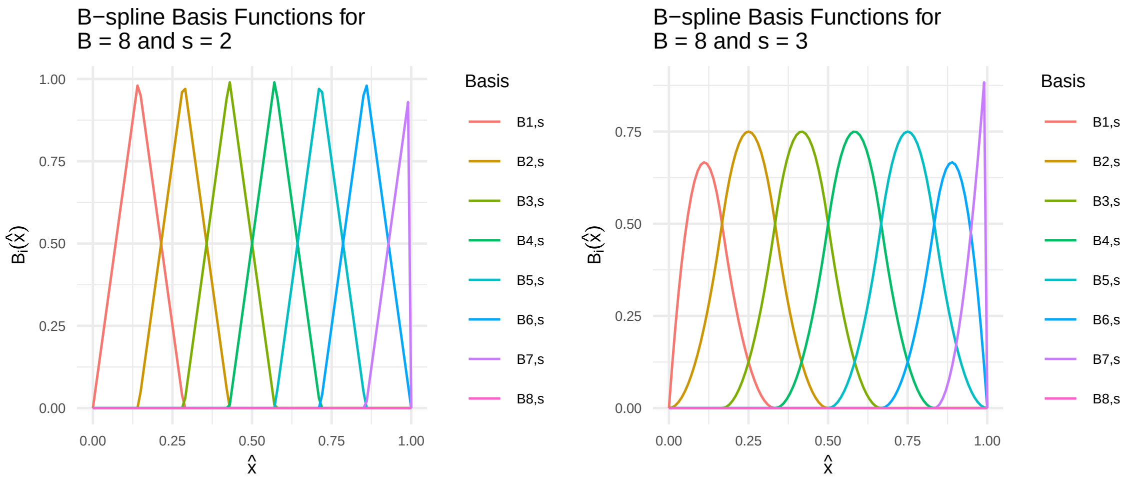

Figure 2: Illustration of B-spline basis functions with different spline order.

Joint probabilities for two and three variables are respectively given as:

$$p(\mathcal{X}_i, \mathcal{Y}_j) = \frac{1}{N} \sum_{v=1}^{N} B_{i,s}(\hat{x}_v) \times B_{j,s}(\hat{y}_v)$$

and

$$p(\mathcal{X}_i, \mathcal{Y}_j, \mathcal{Z}_k) = \frac{1}{N}\sum_{v=1}^{N} B_{i,s}(\hat{x}_v) \times B_{j,s}(\hat{y}_v) \times B_{k,s}(\hat{z}_v)$$

CMI can then be estimated using information theory principles, i.e.,

$$\begin{aligned}
I(X;Y \mid Z) &= H(X \mid Z) - H(X \mid Y, Z) \\
&= H(X, Z) + H(Y, Z) - H(X, Y, Z) - H(Z) \\
&= \sum_{i,k=1..B} p(\mathcal{X}_i, \mathcal{Z}_k) \log_2 p(\mathcal{X}_i, \mathcal{Z}_k) + \sum_{j,k=1..B} p(\mathcal{Y}_j, \mathcal{Z}_k) \log_2 p(\mathcal{Y}_j, \mathcal{Z}_k) \\
&- \sum_{i,j,k=1..B} p(\mathcal{X}_i, \mathcal{Y}_j, \mathcal{Z}_k) \log_2 p(\mathcal{X}_i, \mathcal{Y}_j, \mathcal{Z}_k) - \sum_{k=1..B} p(\mathcal{Z}_k) \log_2 p(\mathcal{Z}_k)
\end{aligned}$$

This method also applies to two or more conditioned variables. The obtained MI or CMI is normalized by $\max(I(X;X), I(Y;Y))$ to have a maximum value of 1 and a minimum value of 0.

1.3 Statistical significance assessment for matrix-vector data format

The main text provides the statistical significance assessment procedure for the case of matrix-matrix input. Here, the statistical significance assessment procedure for the case of matrix-vector input is provided. The input matrix contains rows representing features such as mutations, SCNAs, or cell fractions and columns representing samples. The input vector represents a sample-level variable, such as a gene signature, aneuploidy score, or other cancer hallmark score.

In the matrix–vector setting, the null hypothesis is that a specific input matrix feature is not associated with the input vector, given the variables being conditioned upon. `conMItion` generates an empirical null distribution using a permutation-based randomization procedure. A null distribution of D CMI values is generated similarly to the matrix–matrix setting: in each of D iterations, one vector is independently generated from the matrix by randomly selecting one entry per sample column, and then randomly permuting the result. CMI is calculated between this randomly constructed vector and the original, non-permuted input vector (the variable(s) being conditioned upon also preserve their original order). This preserves the dependence structure between the input vector and the variable(s) being conditioned upon, while disrupting the associations between those two vectors and the input matrix. The empirical P value is defined as the proportion of values from this null distribution that are greater than or equal to the observed CMI. D is typically chosen to be a large value (such as 10^7), permitting the detection of empirical P values as low as D^{-1}.

Similarly, `conMItion` also supports a conditional permutation scheme designed to better preserve the dependence between the randomly sampled vector and the condition variable in matrix-vector input. Under this option, the condition variable is first divided into bins, and the sampled vector is permuted only within each bin rather than across all samples. This within-bin permutation preserves the local structure between the sampled vector and the condition variable while disrupting the association between the sampled vector and the input vector.

The statistical significance of an observed MI value is assessed using the same permutation-based procedure, with the only difference being that no input variables are conditioned upon during calculation. This applies to both the matrix-matrix input and the matrix-vector input.

2 Data pre-processing

2.1 Bladder cancer data

`conMItion` was applied to Urothelial Bladder Carcinoma (BLCA) samples from TCGA to identify interchromosomal SCNA–expression associations. Harmonized HTSeq-FPKM expression data and SCNA data generated by the Affymetrix SNP 6.0 platform were downloaded via TCGAbiolinks [2]. Roughly 54,000 genes were downloaded (detailed gene information can be accessed at `https://github.com/GJYWang/conMItion`). We removed genes that were not expressed in more than 50% of samples, resulting in 29,353 genes. The mutation data for 20,724 genes were obtained from the Multi-Center Mutation Calling in Multiple Cancers (MC3, [3]). The total number of missense mutations for all genes was used to represent mutation burden. To reduce computation time, 40 adjacent genes within the same chromosome arm, or remaining genes at the end of a chromosome arm, were merged into segments, resulting in 1,368 fragments. The SCNA level of a segment was calculated as the average SCNA of its genes. Tumor purity data for BLCA samples were obtained from a recent publication [4]. A total of 393 BLCA samples with expression, mutation, SCNA, and tumor purity information were analyzed. Because the analysis was performed within a single TCGA cancer cohort, we used the expression[1] and SCNA data[2] without additional batch correction. The mutation data and tumor purity estimates used in this study had undergone batch-effect correction [3, 4, 5].

2.2 Single cell RNA-sequencing dataset of lung cancer

Six single-cell RNA sequencing (scRNA-seq) datasets of lung cancer, sequenced using the 10X Genomics platform, were sourced from the Curated Cancer Cell Atlas [6]. Additional datasets were obtained from repositories including the Gene Expression Omnibus (GSE148071), the Sequence Read Archive (PRJNA634159, PRJCA001731, PRJNA622993, PRJNA1055415), and the ArrayExpress database (E-MTAB-6149, E-MTAB-6653). Seurat [7] was used to process all scRNA-seq data, applying dataset-specific quality control thresholds for metrics such as the number of detected genes and the total number of cells. Integration across datasets and batch correction were performed using Harmony. Cell type annotation was performed through the detection of canonical marker genes (see below). The number of cells for each cell type was quantified for all samples, and cell fraction vectors were computed by normalizing cell counts by by the total number of cells per sample. Tumor purity was defined as the malignant cell fraction per sample.

Cell type annotation was performed as follows: NK cells were identified based on the expression of KLRC1, KLRD1, and NKG7; $CD8^+$ T cells were annotated using CD3D, CD3E, CD3G, CD8A, GNLY, and GZMA; $CD4^+$ T cells were annotated using CD4; Treg cells were annotated with FOXP3, CTLA4; B cells were defined by MS4A1, CD79A, and CD79B; plasma cells were defined with CD38, SDC1, JCHAIN; fibroblasts were defined by COL1A1, COL1A2, and DCN; endothelial cells were identified by CLDN5 and VWF; myeloid cells except DC were defined by CD14, LYZ, CD68; M1 macrophages were defined by CD64, CD80, CXCL9, CXCL10, CXCL11, CD86, IL1A, IL6, CD40; M2 macrophages were defined by ARG1, ARG2, IL10, CD163, CCL4, CCL13, CCL17, CCL20, MRC1, MSR1; pDC were annotated by IL3RA, LILRA4, CLEC4C; cDC1 were defined with XCR1, CLEC9A; cDC2 were defined with FCER1A, CD1C; malignant cells were identified by NAPSA, TTF1, KRT5, DSG1, TP63; epithelial cells were annotated by using CAPS, SNTN; and mast cells were defined by GATA2, TPSAB1, TPSB2. Unknown cells were excluded from the analysis.

[1]The HTSeq-FPKM expression profiles retrieved through TCGAbiolinks [2] were normalized for transcript length and sequencing quantity.

[2]The TCGA Affymetrix SNP 6.0 copy-number profiles retrieved through TCGAbiolinks [2] were generated using the TCGA copy-number pipeline, which included array-intensity normalization and tangent normalization to reduce systematic technical variation.

3 Parameter settings and sensitivity analysis

3.1 Sensitivity analysis and guidance for selecting bin number and spline order for CMI

The estimation of CMI for continuous variables requires binning the data. In a hard-binning approach, each data point is assigned to a single bin. This can introduce instability when data points lie near bin boundaries, because small changes in the data or bin setting may change the bin assignment of the data point. In contrast, soft-binning allows each data point to contribute to multiple neighboring bins, thereby reducing boundary effects. In `conMItion`, B-spline functions are used to control the soft-binning process. Two parameters are used: the bin size B and the spline order s. The spline order controls the degree of soft binning: higher spline orders distribute each data point across a larger number of neighboring bins.

Although soft binning can improve the stability of MI/CMI estimation, excessively high spline orders may over-smooth the data distribution. Previous work [9] evaluating the impact of spline order on MI estimation showed that performance, measured by F-score, precision, and recall, deteriorates substantially when the spline order exceeds half of the bin number. This is also intuitive: when $s > B/2$, each data point contributes to more than half of all bins, which can attenuate true associations and introduce spurious dependencies. Therefore, our sensitivity analysis is restricted to examining spline order values smaller than half of the bin number.

To evaluate the sensitivity of different parameters, we used the Lung Cancer Cell Composition dataset described in Section 3.2 of the main text. This dataset was selected because it contains a tractable number of pairs of cell fractions. We repeated the same CMI analysis across a grid of bin numbers $B =$ 6, 8, 10, and 12, and spline orders $s =$ 2, 3, 4, and 5, resulting in 16 combinations. For each setting, we identified significant associations (Fig. 3) between cell fractions after conditioning on tumor purity, using an FDR threshold of 0.2.

The significant CMI associations were highly stable across several parameter settings (Fig. 3), particularly for the bin number and spline order pairs (6,2), (8,2), (8,3), (10,3), (10,4), (12,3), (12,4), and (12,5). These settings consistently identified seven biologically interpretable associations: $CD4^+$ T cells vs myeloid cells, $CD8^+$ T cells vs myeloid cells, $CD8^+$ T cells vs $CD4^+$ T cells, cDC1 vs cDC2 cells, cDC2 cells vs M2 macrophages, cDC2 cells vs myeloid cells, and endothelial cells vs fibroblasts. Only one borderline case was observed, where the association between $CD8^+$ T cells and $CD4^+$ T cells shifted above the FDR threshold (FDR=0.21) under the (8,2) setting.

These results indicate that the detected CMI associations are not driven by a single arbitrary parameter choice. The most stable results were obtained when the spline order was approximately one-quarter to one-third of the bin number. For a fixed bin number, increasing the spline order beyond this range tended to increase the number of significant associations (Fig. 3), as observed for the (6,5), (8,4), (8,5), and (10,5) settings. This pattern is consistent with excessive soft binning leading to over-smoothing of the data. Conversely, reducing the spline order below this range tended to miss the M2 macrophage vs cDC2 cell association while instead identifying the myeloid cell vs cDC1 cell association, as observed for the (10,2) and (12,2) settings.

Based on the above sensitivity analysis, the seven reproducible and biologically interpretable associations were defined as a "high-confidence reference set" for further evaluation of bin number selection. Because these associations were derived empirically from stable parameter settings, the associations detected under a given setting and present in the "high-confidence reference set" were termed "recovered reference associations"; reference associations not detected under a given setting were termed "unrecovered reference associations"; and significant associations detected outside the "high-confidence reference set" were termed "additional non-reference associations".

We next evaluated how bin number selection should depend on sample size (Fig. 4). Using the same dataset, we performed a subsampling analysis. From the original dataset, we generated 100 independent subsampled datasets for each sample size, ranging from 20 to 150 samples in increments of 20. Samples were selected without replacement, while preserving the matched cell-composition matrix and malignant cell fraction (tumor purity).

For each subsampled dataset, we repeated the CMI analysis described in Section 3.2 and identified statistically significant associations (FDR<0.2) between cell fractions after conditioning on tumor purity. We evaluated six bin number and spline order combinations: (6,2), (8,2), (8,3), (10,3), (12,3), and (12,4) (Fig. 4). These settings were selected because the spline order was approximately one-quarter to one-third of the bin number.

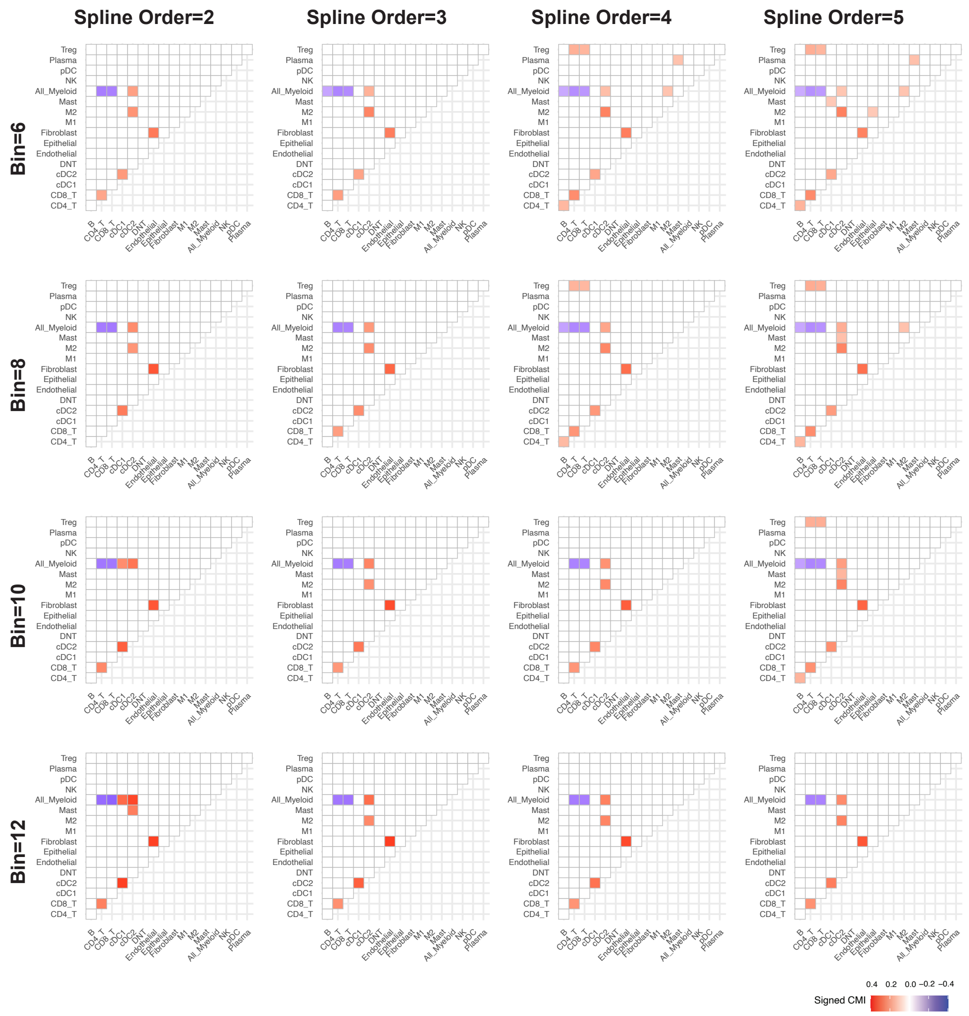

Figure 3: Sensitivity analysis of signed CMI estimates across bin numbers and spline orders. Pairwise cell-type associations in the Cell Fraction of Lung Carcinoma dataset were evaluated using signed CMI under different parameter settings. Rows correspond to different bin numbers (B = 6, 8, 10, and 12), and columns correspond to different B-spline orders (s = 2, 3, 4, and 5). Each panel shows the upper-triangular matrix of signed CMI values between cell-type pairs. Red indicates positive associations, blue indicates negative associations, and color intensity reflects the magnitude of the signed CMI estimate. Only statistically significant associations are illustrated (FDR < 0.2).

To assess the sensitivity of CMI estimation, significant associations from each subsampled dataset were compared with the ”high-confidence reference set” defined above. For each subsampling replicate, we quantified two metrics: the number of “unrecovered reference associations” and the number of “additional non-reference associations”. The average numbers of unrecovered reference associations and additional non-reference associations were then calculated across the 100 subsampling replicates for each sample size and parameter setting.

We summarized the performance of different bin number and spline order pairs across different subsampled sizes (Fig. 4). The number of unrecovered reference associations decreased as the sample size increased, indicating that larger sample sizes improve the recovery of the ”high-confidence reference set”.

This suggests that, within the evaluated range, there is no practical upper limit on the average number of samples per bin.

In contrast, the number of additional non-reference associations decreased as the average number of samples per bin increased (Fig. 4), indicating that a large bin number relative to the sample size can lead to unstable results and the discovery of additional associations. Empirically, the average number of additional non-reference associations decreased below 0.5 (dashed horizontal line in Fig. 4) when the sample size exceeded 30–50 for $B = 6$, approximately 90 for $B = 8$, approximately 110–130 for $B = 10$, and approximately 130 for $B = 12$. These results suggest that reliable CMI estimation requires a sufficient number of data points per bin.

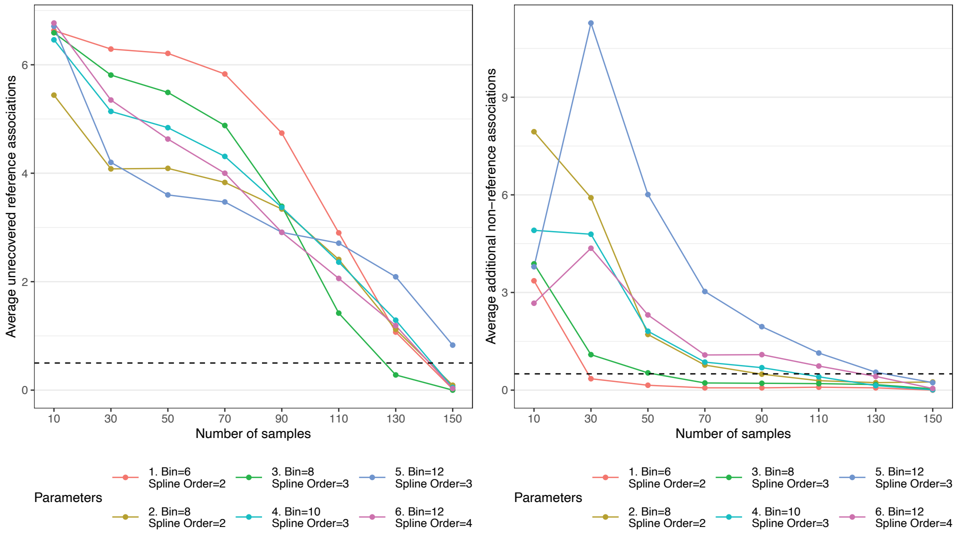

Figure 4: Effect of sample size on recovery of robust reference associations across CMI parameter settings. The stability of CMI-based association detection was evaluated by subsampling the Lung Cancer Cell Composition dataset across different sample sizes: 10, 30, ..., 150. The sampling process was repeated 100 times for each sample size. For each subsampled dataset, associations detected under six bin and spline order combinations – (6, 2), (8, 2), (8,3), (10,3), (12,3), (12, 4) – were compared with the robust reference associations. The left panel shows the average number of unrecovered reference associations, defined as robust reference associations that were not detected in the subsampled analysis. The right panel shows the average number of additional non-reference associations, defined as associations detected in the subsampled analysis but not included in the robust reference set.

In summary, these analyses show that the associations identified by conMItion are robust across a reasonable range of bin numbers and spline orders. We recommend choosing the bin number such that B does not exceed approximately one-tenth of the sample size, corresponding to at least roughly ten samples per bin. Spline order should be selected as a moderate fraction of the bin number, preferably around one-quarter to one-third of B. In the `conMItion` package, the bin number and spline order are automatically selected when users do not specify them. To balance estimation resolution and computational efficiency, the bin number is set to one-tenth of the sample size, with a minimum value of 4 and a maximum value of 12. The spline order is automatically set to the nearest integer to the average of $B/4$ and $B/3$. These restrictions are not applied when users specify their own parameters.

3.2 Impact of bin number and spline order to identifying co-occurring mutation and CNV

We further demonstrate the impact of different parameters on MI/CMI association strength on the TCGA BLCA (Urothelial Bladder Carcinoma) data from Section 3.1 of the main manuscript. The bin numbers are $B = 6$, 8, and 10; and the spline orders are $s = 2$, 3, and 4. We randomly selected 10,000 interchromosomal SCNA-expression pairs from all possible combinations (Fig. 5). The MI between mu-

tation and SCNA, CMI between mutation and SCNA conditioned on tumor purity (termed CMI_p), and CMI between mutation and SCNA conditioned on tumor purity and mutation burden (termed $\mathrm{CMI}_{p,m}$) were estimated (Fig. 5). We observed a strong positive correlation among the association values obtained using different bin numbers and spline orders (Fig. 5).

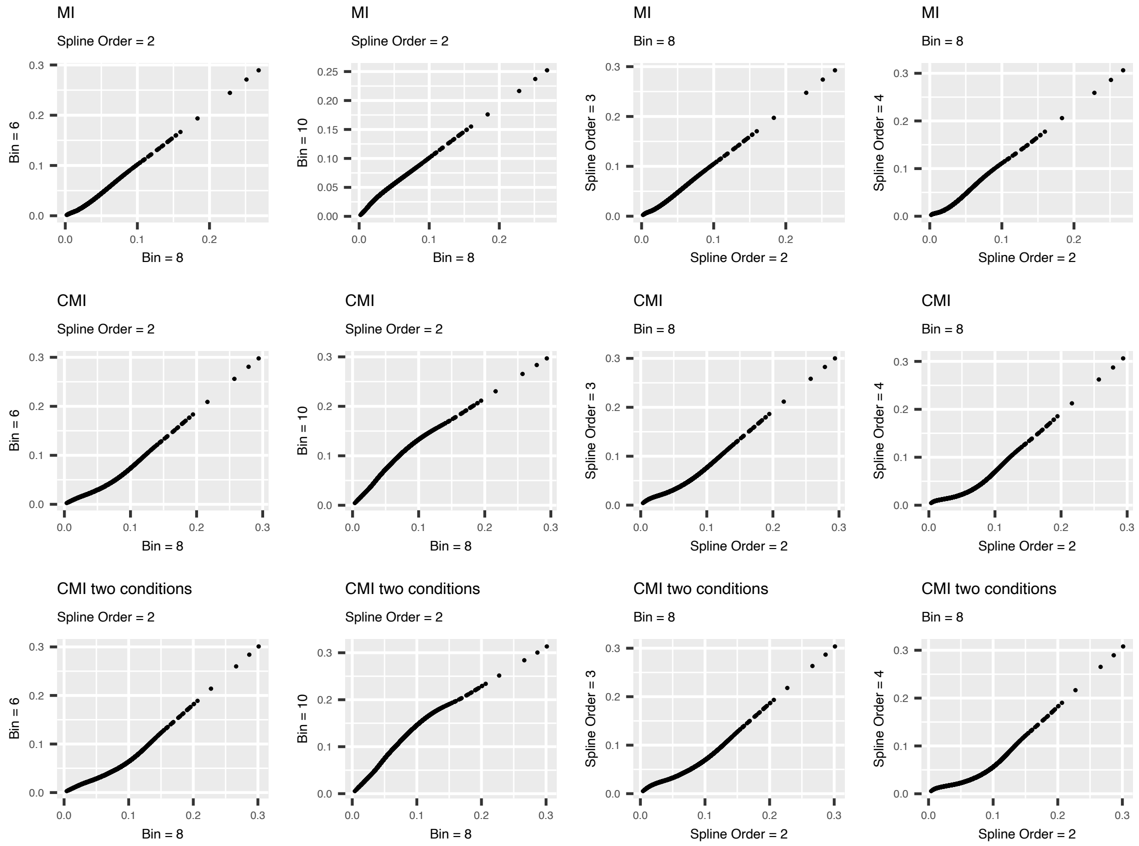

Figure 5: Impact of bin number and spline order on association strength for randomly selected mutation-SCNA pairs. 10,000 mutation-SCNA pairs were randomly selected. MI, CMI_p, and $\mathrm{CMI}_{p,m}$ were evaluated between these 10,000 randomly selected pairs.

4 Comparison between MI/CMI and correlation/linear regression

The motivating question to be addressed in this section is: what are the unique advantages provided by using mutual information (MI) and conditional mutual information (CMI) for computing association between two random variables X and Y, as opposed to computing Pearson/Spearman correlations or using linear regression methods such as ordinary least squares? What differences exist amongst the outputs from both methods?

4.1 Definitions

Ordinary least squares (the most common method for solving linear regression) seeks to determine the optimal coefficients β_0, β_1 satisfying the equation $Y = \beta_0 + \beta_1 * X$ that minimize the total least squares error.

Meanwhile, Pearson correlation between X, Y is defined using the formula below:

$$\rho_{X,Y} = \frac{cov(X,Y)}{\sigma_X \sigma_Y} \tag{1}$$

where $cov(X,Y)$ denotes the covariance between X and Y, and σ_X, σ_Y denote the standard deviations of X and Y respectively.

Spearman correlation is defined simply as the Pearson correlation between the rank values of the input variables.

First, we note that ordinary least squares and Pearson correlation are mathematically related to one another. While the values of β_0 and β_1 returned by ordinarily least squares will vary depending upon the scaling of the input variables X and Y, the p value associated with the estimate for β_1 is identical to the p value for the Pearson correlation between X and Y. In fact, if X and Y are standardized to have unit variance, β_1 becomes equal to the Pearson correlation coefficient [10].

Next, consider the case of a third variable Z. For example, Z can represent a confounding variable. Multiple linear regression seeks to determine the optimal coefficients $\beta_0, \beta_1, \beta_2$ satisfying the equation $Y = \beta_0 + \beta_1 * X + \beta_2 * Z$ that minimize the total least squares error.

Partial Pearson correlation between X and Y given Z is defined as follows: let X' represent the residuals from the linear regression $X \sim Z$, and let Y' represent the residuals from the linear regression $Y \sim Z$. In this scenario, partial Pearson correlation is defined as the Pearson correlation between X' and Y'. Similarly, partial Spearman correlation is defined as the Spearman correlation between X' and Y'.

Similar to before, we note that multiple linear regression and partial correlation are mathematically related to one another. The p value for the estimate of β_1 from multiple linear regression is equal to the p value for the partial Pearson correlation between X and Y given Z [11].

Due to the innate mathematical relationship between least squares regression and (partial) Pearson correlation, we will simplify the analysis that follows to studying the differences between MI vs Pearson/Spearman correlations, and CMI vs partial Pearson/Spearman correlations.

4.2 Comparing MI vs Pearson/Spearman correlation

One of the key limiting assumptions of Pearson correlation is that the input data must be normally distributed [10]. MI/CMI methods, on the other hand, do not require this assumption [1]. Furthermore, both Pearson and Spearman correlations are incapable of detecting certain nonlinear relationships. A classic demonstration of this fact is the function $Y = X^2$ (Fig. 6). Pearson and Spearman correlations in this numeric example are exactly 0, whereas the MI between X and Y is calculated to equal 0.868 (p=0.002).

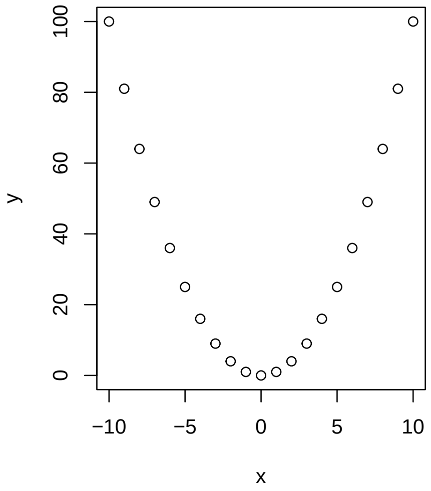

Figure 6: $Y = X^2$ is a classic example of a situation where MI can detect a significant association between X, Y whereas Pearson and Spearman correlation methods do not.

For further examination of the differences observed between MI and Spearman correlation on real-world biological data, please refer to sections 4.4 and 4.5 below, or see [1].

4.3 Comparing CMI vs partial correlation

Now we focus our attention to the comparison between CMI vs. partial correlation methods, both of which estimate the association between X and Y in the presence of a confounding variable Z. Recall that partial correlation between X and Y given Z is, by definition, equivalent to the correlation between the X' and Y' residuals (see section 4.1). Therefore, it follows that in scenarios where the confounding influence of Z is nonlinear, or otherwise not straightforward to regress out simultaneously from both X and Y, that the ability of partial correlation to remove the confounding influence of Z can suffer. Consider the following example in R:

```
set.seed(99)
n <- 150

z <- rnorm(n)
ex <- rnorm(n, sd = 0.5)
ey <- rnorm(n, sd = 0.5)

x <- z + z^2 + ex
y <- -z + z^2 + ey
```

In this example, the confounding influence of Z upon X and Y is nonlinear, since it involves a Z^2 term. The Spearman correlation between X and Y seems to be significant: $\rho = 0.328$ ($p = 4.155 \times 10^{-5}$). The MI between X and Y is similarly significant: MI $= 0.181$ ($p = 2 \times 10^{-5}$).

However, we know that the correlation between X and Y is largely driven by the confounding variable Z. Indeed, the Spearman correlation between $E_X = X - Z - Z^2$ and $E_Y = Y + Z - Z^2$ is $\rho = -0.005$ ($p = 0.955$). The partial Spearman correlation, however, tells a different story:

```
library(ppcor)
pcor.test(x, y, z, method='spearman')
   estimate      p.value statistic   n gp   Method
1 0.4173152 1.192238e-07  5.567661 150  1 spearman
```

The partial Spearman correlation still thinks there is a strong and statistically significant correlation between X and Y ($p = 1.192 \times 10^{-7}$). However, CMI correctly identifies that the relationship between X and Y is no longer statistically significant ($p = 0.706$):

```
library(conMItion)
CMI <- getCMI(x,y,z)
null_dis_cmi <- NULL
CMI
[1] 0.05663874
for (i in 1:1000) {
  null_dis_cmi <- c(null_dis_cmi,getCMI(sample(x,replace = F),y,z))
}
sum(null_dis_cmi>CMI)/length(null_dis_cmi)
[1] 0.17
```

This example demonstrated how partial correlation can fail to remove the confounding influence of Z if the influence of Z upon X and Y is nonlinear. The example that follows next provides yet another example of a situation where partial correlation can fail to remove confounding influence, in the case where the confounding influence of Z is not linearly additive:

```
set.seed(99)
n <- 150

z <- rnorm(n, mean=0, sd=1)
ex <- rnorm(n, mean=0, sd=0.5)
ey <- rnorm(n, mean=1, sd=0.5)

x <- z + ex
y <- z * ey
```

Scatterplots comparing X, Y, and Z are shown below:

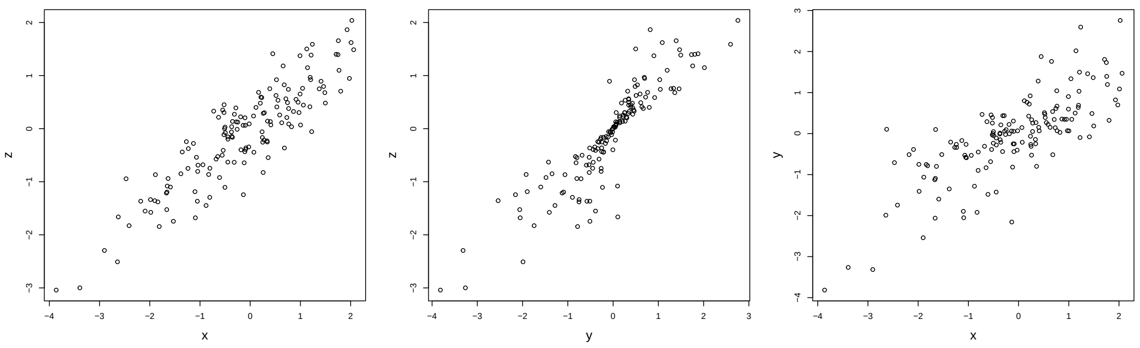

Figure 7: Scatterplots illustrating the relationships between X, Y, and Z

By eye, one can easily appreciate how all three pairs of variables are positively correlated with one another. The Spearman correlation between X and Y is very strong: $\rho = 0.774$ ($p < 2.2 \times 10^{-16}$). The MI between X and Y is similarly strong: MI $= 0.504$ ($p < 10^{-5}$).

However, we know that the correlation between X and Y is largely driven by the confounding variable Z. Indeed, the Spearman correlation between $E_X = X{-}Z$ and $E_Y = Y/Z$ is only $\rho = 0.022$ ($p = 0.788$). The partial Spearman correlation, however, tells a different story:

```
library(ppcor)
pcor.test(x, y, z, method='spearman')
    estimate   p.value statistic   n gp   Method
1 -0.1885816 0.0212647 -2.328204 150  1 spearman
```

Partial Spearman correlation erroneously thinks there is still a statistically significant ($p < 0.05$) correlation between X and Y, albeit in the negative direction. However, CMI correctly identifies that the relationship between X and Y is no longer statistically significant ($p = 0.986$):

```
library(conMItion)
CMI <- getCMI(x,y,z)
CMI
[1] 0.06008561
null_dist <- NULL
for (i in 1:1000) { # null distribution to compute p-value
  null_dist <- c(null_dist,getCMI(sample(x,replace = F),y,z))
}
sum(null_dist>CMI)/length(null_dist)
[1] 0.995
```

Thus, from the two simulated examples above, we have shown that CMI has advantages comparing to partial correlation in situations where the confounding influence of Z is not linearly additive, or not otherwise straightforward to regress out simultaneously from both X and Y.

4.4 Comparing MI/CMI vs (partial) correlation in lung cancer data

We observe differences in the performance of these methods within biological datasets as well. Consider the lung cancer cell type composition dataset, containing the fractional cell type composition of 154 non-small cell lung cancer (NSCLC) tumor samples. The fractional abundance of 18 different cell types (comprising various tumor, immune, and stromal cells) are quantified through scRNAseq for each of the tumor samples.

Biologically, multiple lines of evidence have demonstrated that tumor cells exhibit profound influence in reshaping the composition and abundance of various immune and stromal cell types within the tumor microenvironment, through secretion of various cytokines, chemokines, growth factors, and other signaling molecules, or by altering the composition of the surrounding extracellular matrix to inhibit the infiltration of effector T cells and promote the infiltration of immunosuppressive tumor-associated macrophages [12, 13]. Therefore, we would expect tumor purity (the fractional abundance of tumor cells within a tumor sample) to represent an important confounding variable within this analysis and may be biologically associated with other cell fractions. As shown in Fig. 8 below, tumor purity is negatively correlated with the abundance of various lymphocytes such as CD4^{+} T cells, CD8^{+} T cells, and NK cells.

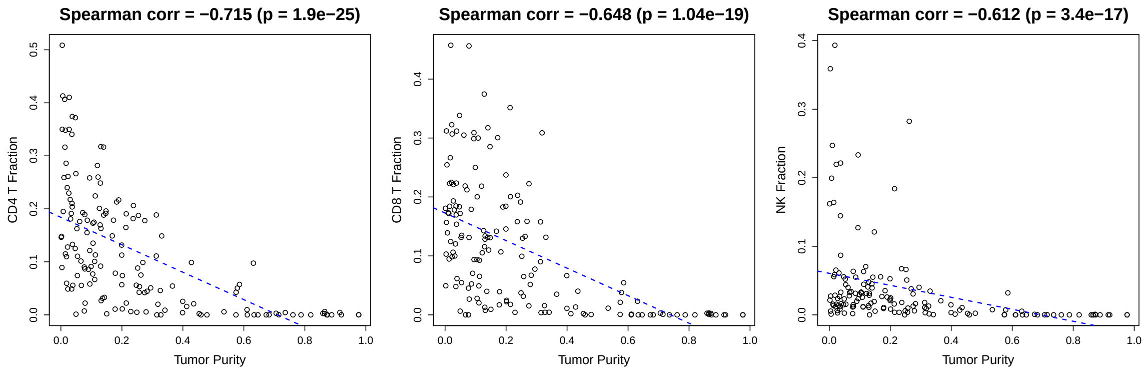

Figure 8: Scatterplots illustrating the negative correlation between tumor purity and the abundance of CD4^{+} T cells, CD8^{+} T cells, and NK cells.

MI/CMI along with (partial) Pearson/Spearman correlations were calculated amongst the compositional fractions of cell type pairs, as shown in Fig. 9. When computing partial correlations and CMI, we conditioned upon tumor purity, a confounding variable present within this dataset.

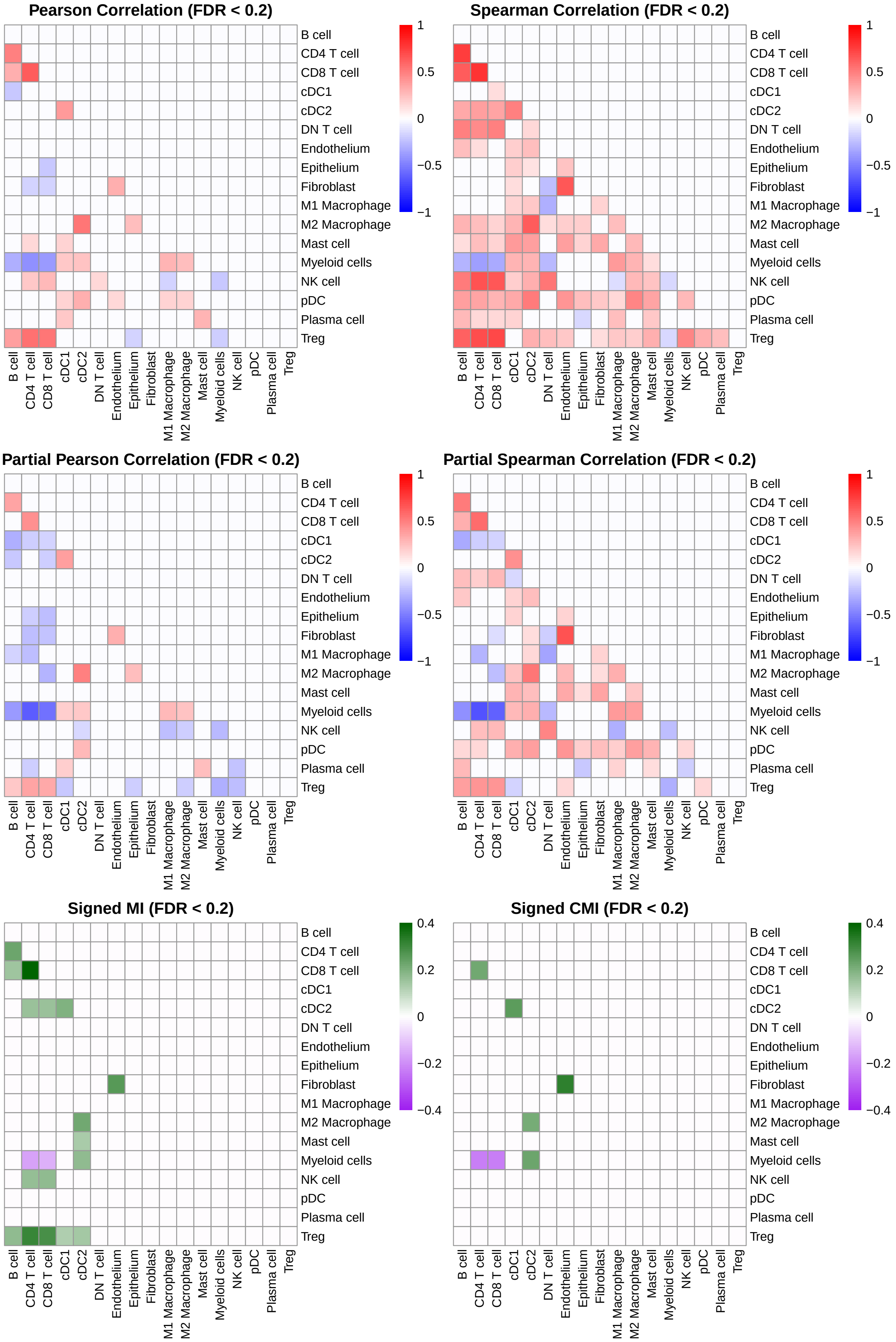

Figure 9: Heatmaps illustrating various measures of association amongst NSCLC tumor sample cell type fractions (FDR < 0.2)

p values were computed for the association between every pair of cell types, and FDR correction was performed within each heatmap using the BH method. Cell type pairs whose association measure had FDR > 0.2 are not shown within each heatmap.

Spearman correlation discovered more statistically significant cell pair associations than Pearson correlation. The significant cell pair associations discovered by CMI are a strict subset of those discovered by MI. That there are fewer statistically significant cell pair associations discovered by CMI compared to MI suggests that some of the cell pair associations discovered by MI may be driven (at least in part) by tumor purity, an external confounding variable.

From the six heatmaps, one can observe that the association between CD8 T cells and Tregs is found to be statistically significant across all metrics except for CMI. To explore why this is the case, observe the scatterplot below of Treg vs CD8 T cell fractions, with tumor purity colored in blue.

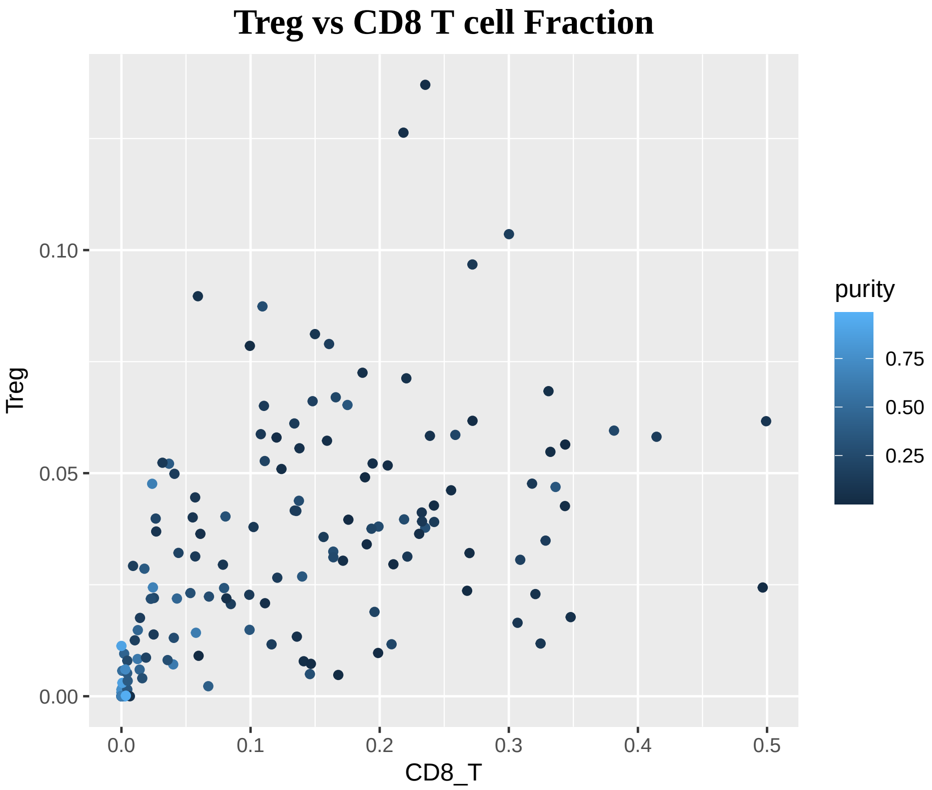

Figure 10: Scatterplot of Treg vs CD8 T cell fractions per NSCLC tumor sample

The Spearman correlation between CD8 T cell fraction and Treg fraction is $\rho = 0.691$ (FDR $= 1.454 \times 10^{-21}$). The MI is similarly strong: MI $= 0.540$ (FDR $< 10^{-6}$). However, one can observe that most of the high-purity tumor samples are clustered in the lower left corner of the scatterplot, and that the large abundance of data points within this region may be artificially inflating the magnitude of association. This can be observed more clearly if we divide the data into two groups: a low purity group (n=77) and a high purity group (n=77) consisting of samples with purity below vs above the median. Shown in Fig. 11 are the scatterplots for data points from each of the two groups separately:

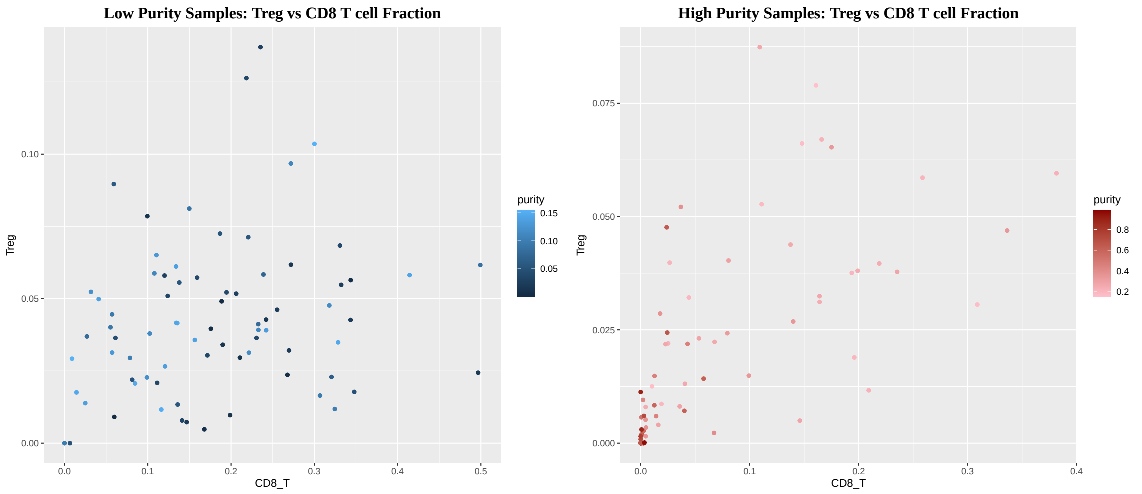

Figure 11: Scatterplot of Treg vs CD8 T cell fractions, divided into low (left) vs high (right) purity samples.

Measures of association for both groups are shown below:

	Low Purity Group	High Purity Group
Spearman corr	0.276	0.849
Spearman p value	0.015	$1.79 * 10^{-22}$
MI	0.205	0.642
MI p value	0.169	$< 10^{-5}$

Table 1: MI & correlation statistics by purity group

As can be concluded both visually from the graph and from the table above, samples in the low purity group exhibit a much weaker positive association/correlation with one another than samples in the high purity group. This example further demonstrates how tumor purity can be a confounding variable affecting the strength of correlation amongst cell type fractions.

Next, we can remove the confounding influence of tumor purity using partial correlation and CMI methods. The partial Spearman correlation between CD8 T cells and Tregs is 0.522 and still statistically significant ($\text{FDR} = 2.920 \times 10^{-5}$). However, the CMI has decreased further to 0.153 ($\text{FDR} = 1$). Thus, this example demonstrates how CMI can be better capable of eliminating the confounding influence of tumor purity, particularly when the influence of the confounding variable is not linearly additive. Furthermore, this result makes intuitive sense biologically, since Tregs are responsible for suppression of immune responses rather than activation of anti-tumor cytotoxicity. Their association with CD8 T cells appears to be a side effect of overall tumor purity.

4.5 Comparing MI vs Spearman correlation in lung cancer data

An interesting pattern observed from the six heatmaps shown in Fig. 9 is that compared to MI, Spearman correlation discovers far more cell type pairs with statistically significant associations ($\text{FDR} < 0.1$). In this section, we explore why this is the case. First, let us consider a small numerical example highlighting a fundamental difference between Spearman correlation and MI. Consider two vectors X, Y defined as

$$X = \{1, 2, 3, 4, 5, 6, 7, 8\}$$
$$Y = \{0, 0, 0, 0, 1, 1, 1, 1\}$$

The Spearman correlation between X and Y is 1, as there is a perfect monotonically increasing relationship between X and Y. However, if X and Y are considered to be discrete random variables whose joint probability mass function is uniformly distributed over each of the eight pairs of values listed, then the MI $I(X;Y)$ normalized with respect to $H(X)$ is only $\frac{1}{3}$. In other words, the MI between X and Y is relatively low because the value of Y (0 or 1) actually does not reveal too much information about the precise value of X. If instead the vector Y had been equal to $\{1,2,3,4,5,6,7,8\}$ as well, then the MI between X and Y normalized with respect to X would have been equal to 1 as well because the value of Y perfectly informs us about the value of X. Hence, this small numerical example provides a concrete illustration of how MI and Spearman correlation do not necessarily go hand-in-hand with one another; a large, statistically significant Spearman correlation between two vectors does not necessarily guarantee that the MI will be similarly large and statistically significant.

Another example that highlights a similar distinction between Spearman correlation vs MI (but using real-world data) can be found in the Waffle Houses dataset [8], which contains data on the number of Waffle House restaurants, median age of marriage, and divorce rates for every state within the United States. The basic premise of this classic statistical problem is that there appears to be a statistically significant Spearman correlation between the number of Waffle House restaurants and the divorce rate across US states (see Fig. 12). However, this correlation is confounded by an external variable: the median age of marriage. Most Waffle House restaurants are located in the southern portion of the United States, where median ages of marriage happen to be lower and divorce rates happen to be higher.

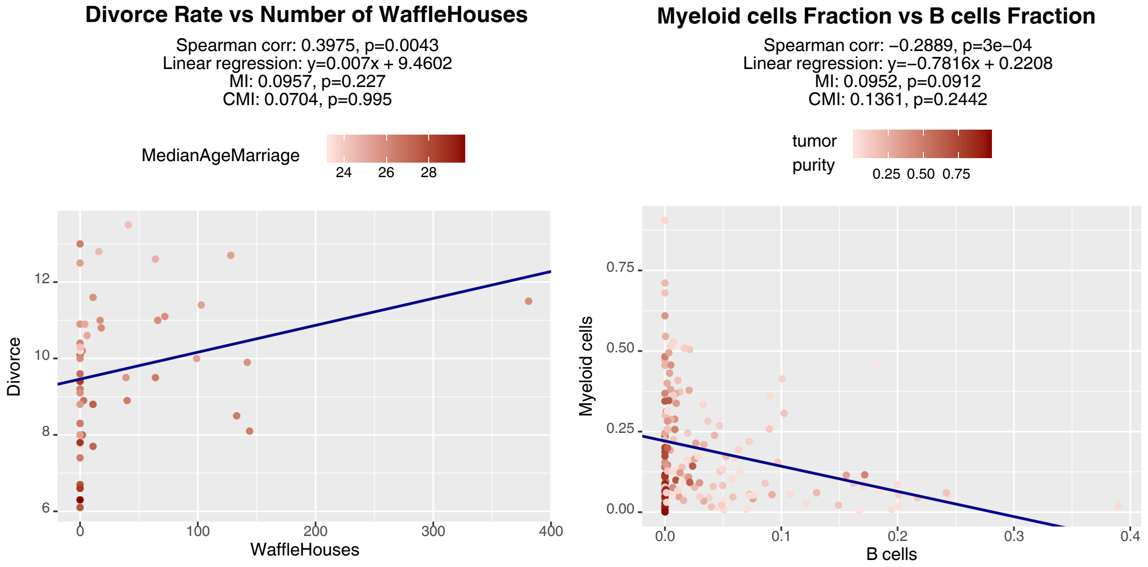

Figure 12: (Left) Divorce rate vs number of Waffle House restaurants, colored by median age of marriage. Each data point represents a US state. (Right) Myeloid cell fraction vs B cell fraction amongst NSCLC tumor samples, with each data point colored by tumor purity.

While the Spearman correlation for this data is moderately large and reaches statistical significance, MI fails to detect any statistically significant association ($p = 0.227$). The absence of statistical significance for MI is likely driven by the abundance of data points lined up along the y-axis, representing states that have zero Waffle House locations but non-zero divorce rates. In the same manner as the small numerical example from above, the abundance of these data points reduces the amount of information which the x-axis variable (number of WaffleHouse restaurants) reveals about the y-axis variable (divorce rate), thereby resulting in a smaller MI that fails to reach statistical significance.

Furthermore, the CMI between divorce rates and number of Waffle House restaurants (conditioned on median age of marriage) has an even smaller magnitude compared to MI and an even larger p value. This is consistent with our prior expectation – that median age of marriage is a confounding variable in the association between divorce rate and number of Waffle House restaurants. This demonstrates how computing CMI with `conMItion` is capable of filtering out spurious correlations driven by external confounding variables.

Returning to the lung cancer tumor microenvironment cell fraction dataset, a similar pattern may help to explain why there are far more statistically significant cell pair associations discovered by Spearman correlation than MI. Similar to the Waffle Houses dataset, the cell composition dataset has a large abundance of 0 entries, representing tumor samples that had 0 counts of a particular cell type. (This often arises in high-purity tumor samples that are predominantly composed of malignant cells). See the right panel of Fig. 12, illustrating the relationship between myeloid cell fraction vs B cell fraction. Similar to the Waffle Houses example, there are many data points lined up against the y-axis, contributing to a Spearman correlation that is statistically significant but MI that is not.

To quantify this pattern more formally across all cell type pairs being examined, consider an arbitrary pair of cell types, cell type i and cell type j, with corresponding cell fraction vectors F_i and F_j. F_i and F_j have length equal to the number of tumor samples. For every sample s, we can define the tuple (F_{is}, F_{js}) to represent the fraction of cell type i and j found in sample s. For each cell type pair (i, j), we can define its number of *unique intercepts* as the number of unique sample tuples satisfying the form $(0, F_{js})$ where $F_{js} \neq 0$ or $(F_{is}, 0)$ where $F_{is} \neq 0$. We hypothesized that, just as we saw in the small numerical example and Waffle Houses example from above, cell type pairs which fail to achieve statistically significant MI are more likely to have a greater number of unique intercepts.

Shown below is a scatterplot containing 136 data points (corresponding to 136 unique cell type pairs), where the x-axis plots the FDR value from the Spearman correlation amongst the cell type fractions for this pair, and the y-axis plots the FDR value from the MI amongst the cell type fractions for this pair. Furthermore, each data point is colored by its number of unique intercepts. Dashed horizontal and dashed vertical lines are located at $x = 0.1$ and $y = 0.1$ to indicate the FDR thresholds (see Fig. 13A).

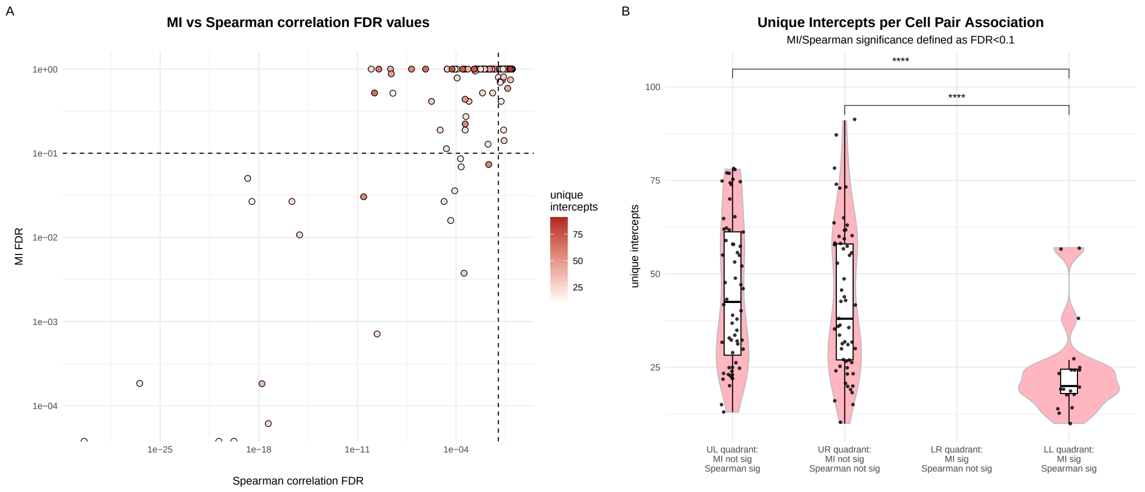

Figure 13: (A) MI vs Spearman correlation FDR values for each unique cell type pair, colored by its corresponding number of unique intercepts. (B) Violin plot showing distributions for number of unique intercepts per cell pair association, grouped by quadrant.

The location of data points in Fig. 13A can be interpreted as follows:

- Upper left (UL) quadrant: cell pair associations that are significant according to Spearman but not according to MI
- Upper right (UR) quadrant: cell pair associations that are not significant according to both methods
- Lower right (LR) quadrant: cell pair associations that are significant according to MI but not according to Spearman
- Lower left (LL) quadrant: cell pair associations that are significant according to both methods

Interestingly, there are no data points in the lower right quadrant, indicating that the statistically significant cell pair associations identified by MI are a strict subset of those identified by Spearman.

Most of the dark red points (cell pairs with high numbers of unique intercepts) are located in the upper left and upper right quadrants, but this pattern can be examined more thoroughly in the violin plot shown in Fig. 13B. The cell pair associations that are classified as significant by both MI and Spearman (LL quadrant) have significantly fewer unique intercepts than cell pairs in the UL quadrant and cell pairs in the UR quadrant. Overall, this result is highly consistent with the intuition built from the previous three examples – that an abundance of data points where one random variable is held constant while the other random variable varies reduces the magnitude and statistical significance of MI.

5 Runtime analysis

To evaluate the computational cost of generating null distributions, we evaluated the runtime of MI and CMI estimation using two datasets from the main manuscript: the TCGA BLCA dataset from Section 3.1 (393 samples) and the Cell Fraction of Lung Carcinoma dataset from Section 3.2 (154 samples).

Runtime was assessed for MI and CMI estimation under three parameter settings: bin number and spline order pairs of (6,2), (8,2), and (12,4). For each parameter combination, we performed permutation-based testing (Section 2.3 in the main text) for three types of association: MI, CMI conditioned on tumor purity (for both the TCGA BLCA dataset and the Cell Fraction dataset), and CMI conditioned on both tumor purity and mutation burden (only for TCGA BLCA dataset).

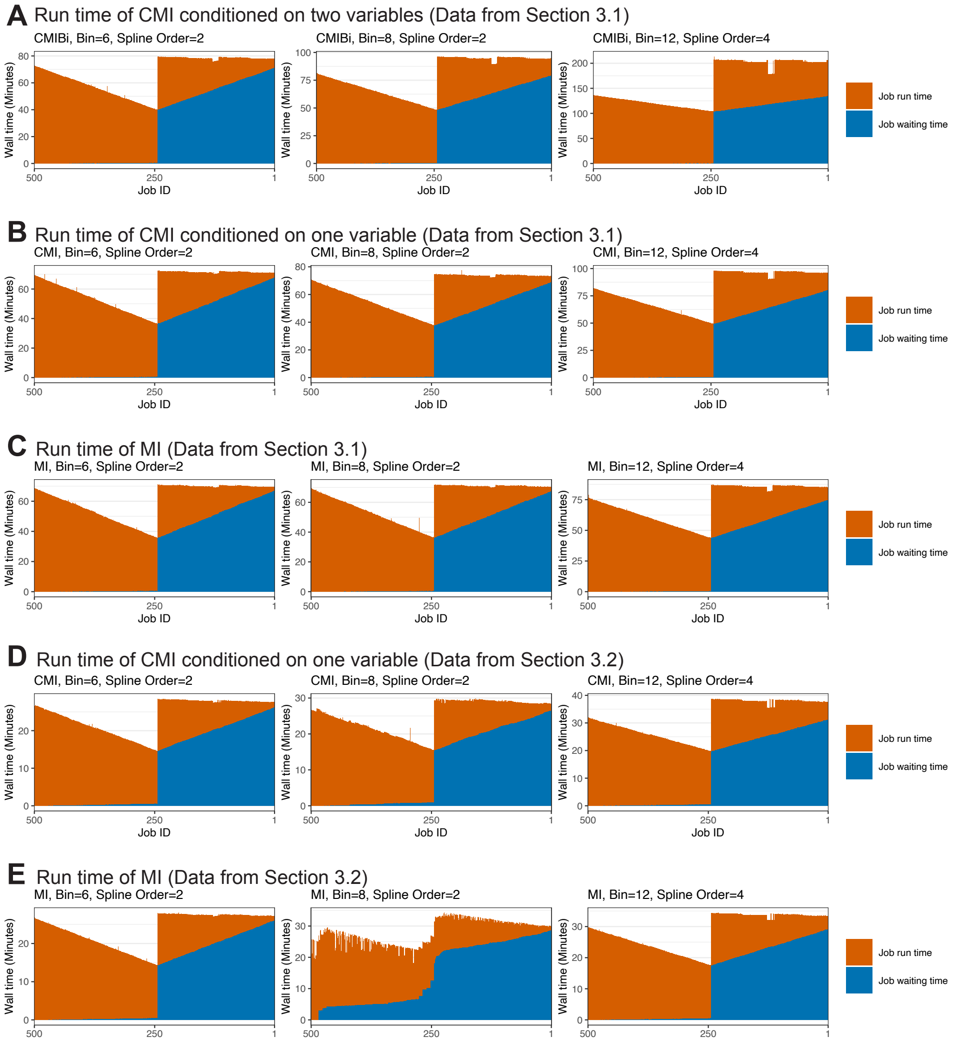

Figure 14: Runtime evaluation for generating null distributions using datasets from Sections 3.1 and 3.2 of the main manuscript. For each association measure and parameter setting, 100,000,000 permutations were divided into 500 independent jobs, with 200,000 permutations assigned to each job. Stacked bars show the wall time for each job, decomposed into job waiting time and actual job runtime. Jobs are ordered by job ID on the x-axis, and wall time is shown in minutes on the y-axis. (A) Runtime of CMI conditioned on two variables using the dataset from Section 3.1. (B) Runtime of CMI conditioned on one variable using the dataset from Section 3.1. (C) Runtime of MI using the dataset from Section 3.1. (D) Runtime of CMI conditioned on one variable using the dataset from Section 3.2. (E) Runtime of MI using the dataset from Section 3.2. For each analysis, three representative parameter settings were evaluated: bin number and spline order pairs of (6,2), (8,2), and (12,4).

For each association measurement and parameter pair, the permutation analysis was divided into 500 independent jobs. In total, 100,000,000 permutations were calculated for each setting, corresponding to

200,000 permutations per job. Each job was submitted to the high-performance server “wallaby”, a Dell PowerEdge R7525 system equipped with 256 AMD EPYC 7763 CPU cores and 1031.3 GB RAM, running a 64-bit Linux environment. Jobs were submitted in descending order of expected runtime, starting with the longest running jobs and ending with the shortest running jobs (Fig. 14). The submission time, start time, and end time of each job were recorded, allowing us to quantify queue waiting time, actual computational runtime, and total wall time.

For each job, the computational runtime was calculated as the difference between the recorded start and end times. The waiting time was calculated as the difference between the first submission time and the recorded start time of each job. These quantities were visualized as stacked bar plots, allowing the total wall time to be decomposed into queue waiting time and actual computation time. The implementation of the conMItion package enables the generation of null distributions based on permutations to be parallelized across independent jobs. This avoids a single long serial computation and allows large-scale significance testing to be efficiently distributed across multiple CPU cores or compute nodes.

Overall, the runtime analysis showed that large-scale permutation is computationally feasible when parallelized. Runtime increased with spline order and with the number of conditioning variables. The longest runtime was observed for the most computationally demanding setting: CMI conditioned on two variables, using a bin number of 12 and spline order of 4, in the TCGA BLCA dataset from Section 3.1. Under this setting, the total runtime was approximately 200 minutes (Fig. 14A). With a smaller bin number and spline order, such as (8,2), the total runtime decreased substantially. For CMI conditioned on two variables (Fig. 14A), CMI conditioned on one variable (Fig. 14B), and MI (Fig. 14C), the corresponding runtime was approximately 100, 70, and 70 minutes, respectively.

Runtime was further reduced in the lung cancer cell type composition dataset from Section 3.2, which contains fewer samples than the TCGA BLCA dataset (154 vs 393 samples). Even under the largest parameter setting, (12,4), the total runtime for CMI conditioned on one variable (Fig. 14D) and MI (Fig. 14E) was approximately 40 and 35 minutes, respectively.

Because the analysis was distributed over 256 CPU cores, each core processed approximately two jobs on average. In principle, the wall time can be further reduced on systems with more available CPU cores, provided that the number of jobs is sufficiently large. For example, if 500 jobs are distributed across 500 available CPU cores, the total wall time would be expected to be the runtime of the longest individual job, excluding queueing and scheduling time. Most importantly, we used an extremely large null distribution of 100 million permutations in this analysis. A run with 10 million permutations would take roughly 20 minutes.

6 Associated mutation-SCNA pairs in BLCA

In this section, we used mutation–SCNA analysis in BLCA to show that `conMItion` can prioritize candidate associations while accounting for tumor purity and mutation burden and to illustrate the practical limitations of permutation-based significance testing when mutation data are sparse.

`conMItion` was applied to BLCA samples to identify co-occurring or mutually exclusive missense mutations and SCNAs. The mutation data for 20,724 genes were obtained from the Multi-Center Mutation Calling in Multiple Cancers (MC3, [3]). We also used the SCNA levels for 1,368 genomic segments that together comprise the whole genome (Supplementary Methods). Across 393 patient samples, `conMItion` estimated the CMI between the mutation status of 20,724 genes and the SCNAs of 1,368 genomic segments (approximately 28 million mutation-SCNA pairs) while conditioning on tumor purity and mutation burden (termed $\mathrm{CMI}_{p,m}$), using a bin number of 8 and a spline order of 2 (Supplementary Document). Due to the sparsity of the mutation data, mutation had substantially lower estimated entropy than expression and SCNA (Figure 15).

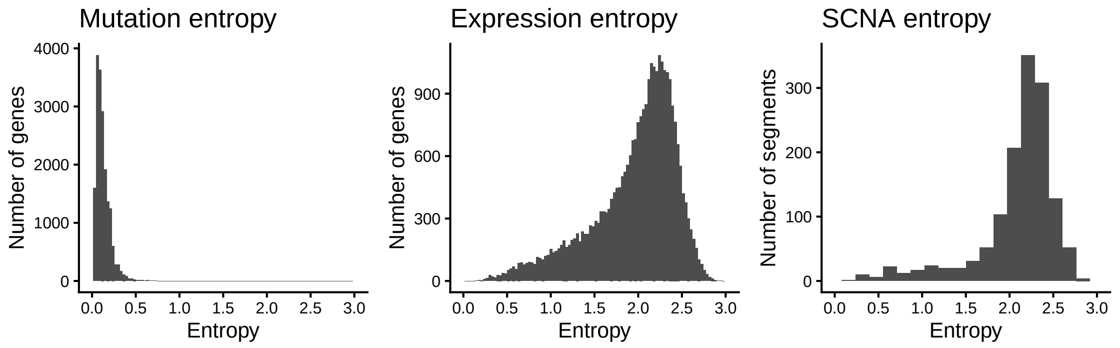

Figure 15: Entropy distributions of mutation, expression, and SCNA. Histograms show the entropy values of mutation, expression, and SCNA in the BLCA data. Due to the sparsity of mutation data, mutation entropy is highly concentrated near 0.2. In contrast, expression and SCNA features show substantially higher entropy. The entropy values were estimated with a bin number of 8 and a spline order of 2. The upper limit of the entropy value is 3.

Because mutation data were sparse, most genes were mutated in only a small number of samples. During permutation, the sampled vector from the mutation matrix also contained a small number of mutated samples. To examine whether the permutation-based procedure generated mutation vectors similar to one of the gene mutations, we compared the randomized and permuted mutation vectors with the original mutation matrix. For each permutation, one entry was randomly sampled from the mutation matrix for each sample, producing a randomized mutation vector with the same sample dimension as an original mutation data; this vector was then permuted across samples (Section 2.3 in the main text). For each permuted vector, we calculated the mutation count, i.e. the number of mutated samples, and the minimum Hamming distance to any row of the original mutation matrix.

Permuted vectors with low mutation counts showed very small Hamming distances to original mutation features, indicating that sparse null vectors can resemble real low-frequency mutation profiles (Figure 16). The mutation counts of the permuted vectors were also concentrated at values below 10 (Figure 16), showing that such sparse vectors were frequently generated. These random and permuted vectors resembling actual mutation features may lead to limited separation between observed MI/CMI values and the null distributions. The observed CMI distribution was highly concentrated near zero and closely resembled the null distribution (Figure 17). This limited the ability of the permutation test to produce sufficiently small empirical P values. Together with the large number of mutation–SCNA pairs tested, all FDR values were close to 1, and no association remained statistically significant after Benjamini–Hochberg (BH) correction. This likely reflects the combined effects of sparse mutation data, limited information (entropy) for most mutations, and the large number of tested mutation–SCNA pairs.

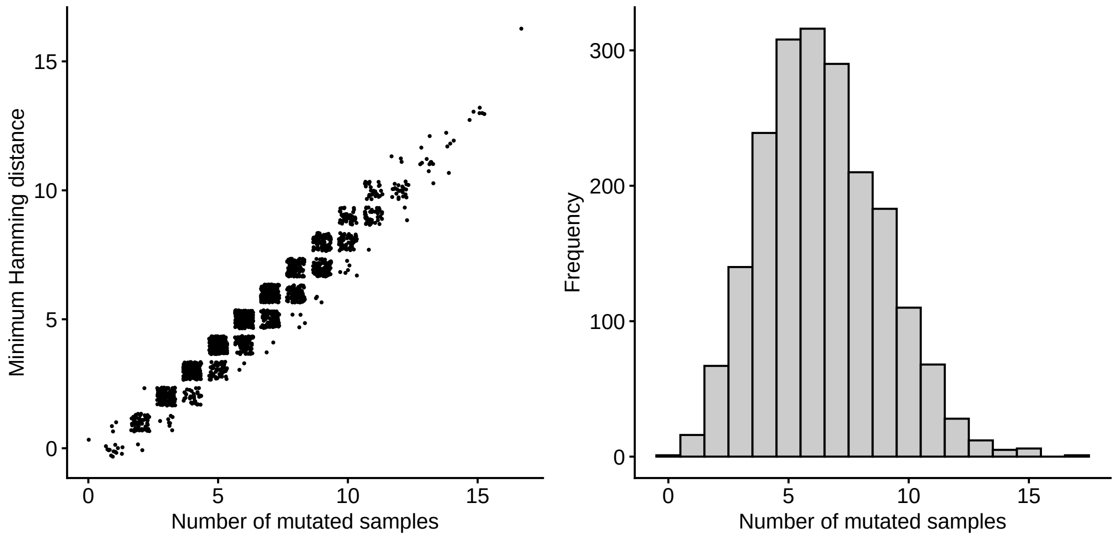

Figure 16: Permuted mutation vectors can resemble original mutation features. For each randomly sampled and permuted mutation vector, we calculated the number of mutated samples and the minimum Hamming distance to any mutation feature in the original mutation matrix. (Left) Vectors with fewer mutated samples showed smaller minimum Hamming distances, indicating that sparse null vectors can closely resemble real low-frequency mutation profiles. (Right) The histogram shows the distribution of mutation counts among permuted vectors, with most vectors containing number of mutated samples lower than 10.

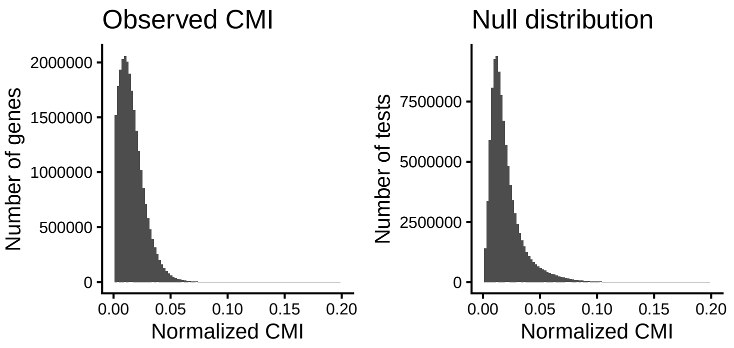

Figure 17: CMI distribution of all mutation–SCNA pairs and the null distribution from permutation.

Next, we performed an exploratory analysis with a significantly reduced number of hypothesis tests. We evaluated whether biologically interpretable mutation–SCNA relationships could be recovered when the number of tests was reduced and mutation data were restricted to frequently mutated genes. In this modified analysis, copy number was averaged across all genes per chromosomal arm (e.g. 1p, 1q, 2p, 2q, etc.) producing average copy number values for each sample across a total of 44 chromosomal arms. The top 50 most frequently mutated genes across all BLCA samples were selected. This resulted in a total of $44 \times 50 = 2200$ hypothesis tests conducted, significantly fewer than the original $1368 \times 20724 = 28350432$ tests. Association values were signed based upon the sign of the corresponding Spearman correlation, see Figure 18, 19, 20.

One of the strongest associations that stood out from this analysis was the association between *RB1* mutation and chr9p copy number variation. The MI was 0.069 ($p = 3.0 \times 10^{-7}$, FDR $= 2.20 \times 10^{-4}$, rank=3), CMI_p was 0.079 ($p = 6.0 * 10^{-7}$, FDR $= 3.3 \times 10^{-4}$, rank=3), and $\text{CMI}_{p,m}$ was 0.085 ($p = 6.0 \times 10^{-7}$, FDR $= 2.64 \times 10^{-4}$, rank=5). Returning to the previous analysis, in which 28 million hypothesis tests were performed, we found that the association between *RB1* mutation and the 9:21.5-25.8Mb genomic region had a CMI_p value of 0.108 ($p = 7.87 \times 10^{-4}$, rank=172), and $\text{CMI}_{p,m}$ value of

0.109 ($p = 7.89 \times 10^{-4}$, rank=256).

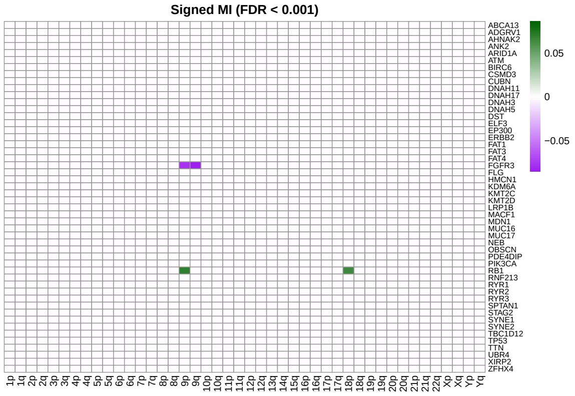

Figure 18: Signed MI (FDR < 0.001) amongst 50 most frequently mutated genes in BLCA and chromosomal arm copy number variation.

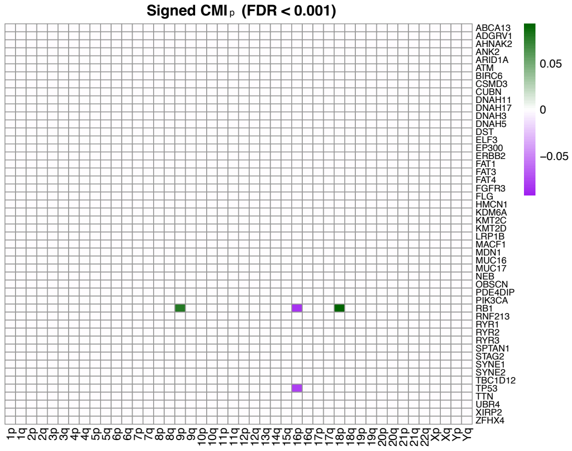

Figure 19: Signed CMI_p (FDR $<$ 0.001) amongst 50 most frequently mutated genes in BLCA and chromosomal arm copy number variation.

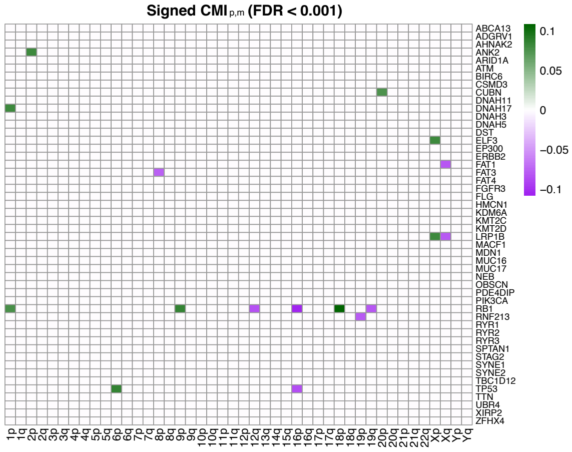

Figure 20: Signed $\mathrm{CMI}_{p,m}$ (FDR $<$ 0.001) amongst 50 most frequently mutated genes in BLCA and chromosomal arm copy number variation.

This association makes biological sense. *RB1* mutation and chr9p loss might be mutually exclu-

sive (Figure 21) because they are functionally redundant and offer similar proliferative advantages to cancer cells. The 9:21.5-25.8Mb genomic region contains the *CDKN2A* and *CDKN2B* tumor suppressors, whose loss frees the cyclin D : CDK4/6 complex to promote cell cycle progression past the G1/S checkpoint. *RB1* mutation accomplishes the same outcome through ineffective binding/sequestration of the downstream E2F transcription factor. In this analysis, `conMItion` recovered a biologically plausible mutation-SCNA association. The absence of FDR-significant associations in the full mutation–SCNA analyses should therefore be interpreted as reflecting limited statistical power with sparse mutation data and correction for a large number of tests, rather than as evidence that mutation–SCNA associations are absent in BLCA.

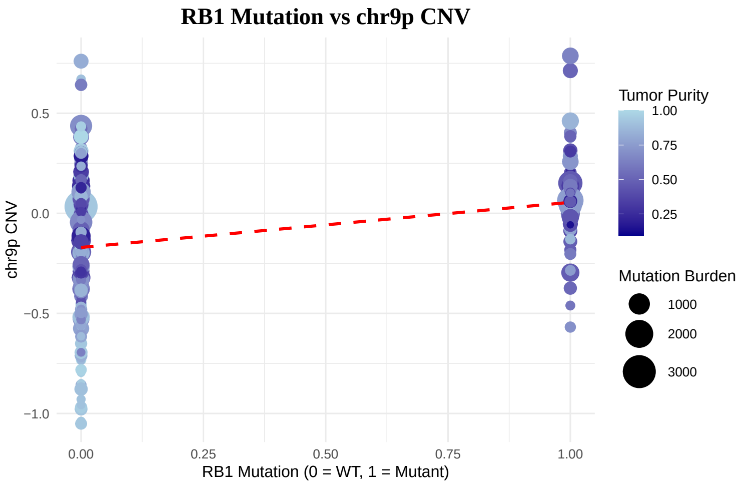

Figure 21: Plotting association between *RB1* mutation and chr9p copy number variation in bladder cancer.

The magnitude and rank of MI/CMI values, together with the comparison between MI and CMI, remain useful for prioritizing candidate associations. The 374 mutation–SCNA pairs with the highest CMI values had empirical $P < 10^{-3}$ (Table 2). TP53 mutations demonstrated an association with 16p13.3 SCNAs (rank = 169), which contains the gene E4F1, a transcription factor known to interact with TP53 [14]. In this region, 29.0% of BLCA samples showed deletions smaller than -0.2 (log2 ratio). 21.4% of BLCA samples acquired both TP53 mutations and E4F1 deletions. Although this association did not survive BH correction, it shows that CMI rankings can still prioritize biologically interpretable candidates.

Furthermore, `conMItion` evaluated changes in association strength for mutation–SCNA pairs accounting for different confounding factors. For comparison, we further calculated MI without conditioning and CMI conditioned only on tumor purity (CMI_p). The association between GINS3 missense mutations and SCNAs at 2q34, containing ERBB4, was stronger when overall mutation burden was not controlled (MI = 0.089, rank = 125, $P = 5.4 \times 10^{-4}$; $\text{CMI}_p = 0.065$, rank = 23,660, $P = 1.8 \times 10^{-2}$). However, $\text{CMI}_{p,m}$ for this mutation-SCNA pair was 0.028 (rank = 3,445,005, $P = 0.20$), suggesting the magnitude of the association was inflated by mutation burden as a confounding variable. Similarly, the association between FGFR3 missense mutations and SCNAs at 9q21.33 (MI = 0.087, rank = 167, $P = 6.2 \times 10^{-4}$) underwent a decrease in magnitude after conditioning on tumor purity ($CMI_p = 0.046$, rank = 214,730, $P = 0.055$), suggesting that the observed MI association for this mutation-SCNA pair could be inflated by tumor purity. These examples demonstrate how `conMItion` can assess whether the observed associations are inflated by confounding factors though direct comparison between normalized MI and CMI values.

These results demonstrate a practical limitation of applying permutation-based significance testing to sparse mutation matrices. In the full mutation–SCNA analyses, sparse mutation profiles and the large testing burden prevented any pair from surviving BH correction. Nevertheless, MI/CMI estimates remain informative for ranking candidate associations and, more importantly, for evaluating whether apparent mutation–SCNA associations are attenuated after conditioning on tumor purity and mutation burden.

Table 2: Top mutation–SCNA associations ($P<0.001$) identified by CMI after accounting for tumor purity and mutation burden.

Mutation	SCNA	CMI	P Value	Mutation	SCNA	CMI	P Value	Mutation	SCNA	CMI	P Value
RPS6	1:76.7-78.7Mb	0.15539445	0.0000303	DOCK9	19:7.9-8.8Mb	0.11475019	0.000529	T	1:117.9-119.9Mb	0.10935751	0.0007785
MTA1	12:10.6-11.5Mb	0.15471906	3.186E-05	C7orf43	21:16.6-19.3Mb	0.11474612	0.0005292	ARMC5	19:29.5-32.5Mb	0.10935647	0.0007785
DONSON	12:10.6-11.5Mb	0.1530079	3.566E-05	GRM8	12:55.4-56Mb	0.1146699	0.0005323	RABGGTA	13:48.3-50Mb	0.10927856	0.0007829
C1orf127	12:10.6-11.5Mb	0.14964844	4.574E-05	ZNF7	19:27.6-29.5Mb	0.1145852	0.0005355	RB1	16:0.9-1.4Mb	0.10926196	0.0007838
HNRNPA2B1	1:121.6-121.6Mb	0.14701593	5.495E-05	RRAGB	1:206.1-207.3Mb	0.11458413	0.0005356	TP53	16:2.6-3.1Mb	0.10923858	0.0007852
GPR162	1:121.6-121.6Mb	0.14464142	6.504E-05	ICE1	22:22.8-23.4Mb	0.11450034	0.0005389	SOX5	12:65.8-68.2Mb	0.1092329	0.0007855
ABL1	12:10.6-11.5Mb	0.14359865	6.994E-05	NELL2	1:117.9-119.9Mb	0.11429008	0.0005475	RB1	9:21.5-25.8Mb	0.10916644	0.0007892
HCRTR2	7:112.3-116.3Mb	0.14099778	8.394E-05	ESRP1	2:179.1-182.9Mb	0.11417104	0.0005524	CNTNAP3	1:121.6-121.6Mb	0.10915488	0.00079
MSI2	1:53.9-55.2Mb	0.13821561	0.0001014	ASXL3	17:31.8-32.5Mb	0.11409598	0.0005553	ADAMTS13	1:121.6-121.6Mb	0.10910908	0.0007924
PDE5A	1:121.6-121.6Mb	0.1370824	0.0001103	PARP10	11:54.6-55.7Mb	0.11403623	0.0005576	WDR36	5:114.5-116.6Mb	0.1091007	0.0007929
CEP250	1:119.9-121.6Mb	0.13662377	0.000114	SI	11:99.1-102.8Mb	0.1140252	0.000558	WDFY3	17:31.8-32.5Mb	0.10908797	0.0007937
PRPF4B	1:189.9-193.8Mb	0.13618077	0.0001172	MMP8	12:64.3-65.8Mb	0.11400798	0.0005587	SPATA31D1	1:76.7-78.7Mb	0.10900984	0.0007977
SPEM2	1:206.1-207.3Mb	0.13610375	0.0001178	TGS1	17:31.8-32.5Mb	0.11397135	0.0005601	CNTNAP2	12:9.7-10.6Mb	0.10899719	0.0007984
VWCE	13:48.3-50Mb	0.13590649	0.0001194	DLEC1	1:119.9-121.6Mb	0.11387357	0.0005642	SIGLEC12	12:10.6-11.5Mb	0.10898913	0.0007989
MED12	4:81.3-83.4Mb	0.13567541	0.0001215	DRD3	1:121.6-121.6Mb	0.11380697	0.0005668	ZNF615	1:121.6-121.6Mb	0.10897838	0.0007995
MMP2	17:38.4-39.1Mb	0.13544485	0.0001236	PTPDC1	12:10.6-11.5Mb	0.11374936	0.0005688	TRPM2	1:53.9-55.2Mb	0.10897498	0.0007996
SLC25A40	12:10.6-11.5Mb	0.13474555	0.0001299	ATP6V1A	11:54.6-55.7Mb	0.11373855	0.0005693	NUDT22	2:127.9-130.2Mb	0.10896581	0.0008
GSTM2	1:40.7-42.6Mb	0.13432997	0.0001339	ADGRG1	1:117.9-119.9Mb	0.11372677	0.0005697	LTBP1	1:39.7-40.7Mb	0.10892907	0.0008019
VIL1	1:76.7-78.7Mb	0.13421861	0.0001351	STAG3	1:49.3-51.8Mb	0.11369372	0.0005711	TPGS2	1:119.9-121.6Mb	0.1089273	0.0008021
RHBG	6:89.3-93.1Mb	0.13256121	0.0001524	CAMSAP1	1:121.6-121.6Mb	0.11363692	0.0005734	PWP2	1:119.9-121.6Mb	0.10887443	0.0008054
SORBS3	12:10.6-11.5Mb	0.13227783	0.0001554	KDM1A	22:22.2-22.6Mb	0.11362684	0.0005738	KIDINS220	12:54-54.3Mb	0.10881428	0.0008091
LILRB1	11:54.6-55.7Mb	0.13181407	0.0001603	FYCO1	4:87.3-88.7Mb	0.11352188	0.0005777	BUB1	1:206.1-207.3Mb	0.1088075	0.0008094
NFATC1	1:121.6-121.6Mb	0.12893009	0.0001971	POLR2A	11:54.6-55.7Mb	0.11349187	0.0005789	MTMR11	11:54.6-55.7Mb	0.10874553	0.0008129
SLCO1A2	5:114.5-116.6Mb	0.12837276	0.0002048	ANO8	16:9.6-11.3Mb	0.11347451	0.0005795	KDM1A	22:21.6-22.2Mb	0.1086389	0.0008186
LRRC37A3	12:64.3-65.8Mb	0.12827296	0.000206	ZNF516	17:19.2-19.7Mb	0.11341843	0.0005817	TNK2	1:62.2-63.8Mb	0.10849455	0.0008272
PUS3	1:121.6-121.6Mb	0.1279112	0.0002114	FLG2	3:85.8-90.3Mb	0.11340132	0.0005824	BPTF	7:53.5-55.7Mb	0.10847925	0.0008282
FAM161A	1:121.6-121.6Mb	0.12762971	0.0002152	KDM1A	22:21-21.6Mb	0.11335363	0.0005841	AKT1	11:99.1-102.8Mb	0.10843339	0.000831
CEP250	1:121.6-121.6Mb	0.12712121	0.0002224	UPF2	1:53.9-55.2Mb	0.11328913	0.0005868	IGSF9B	22:22.8-23.4Mb	0.1084203	0.0008317
AZIN1	1:76.7-78.7Mb	0.12705068	0.0002237	GRIK3	1:76.7-78.7Mb	0.11316087	0.000592	HERC2P3	1:76.7-78.7Mb	0.10839413	0.0008335
MSH4	12:55.4-56Mb	0.12692693	0.0002254	AMER3	6:105.1-107.8Mb	0.11314343	0.0005928	HHIPL2	12:10.6-11.5Mb	0.10830887	0.0008387
TPCN1	1:121.6-121.6Mb	0.12676522	0.0002279	DNM1P47	1:117.9-119.9Mb	0.11311542	0.0005942	LY9	3:49.9-50.6Mb	0.10827161	0.0008409
LIMA1	1:121.6-121.6Mb	0.12657313	0.000231	MUC3A	1:121.6-121.6Mb	0.11308392	0.0005951	GTF2A1	13:48.3-50Mb	0.10820103	0.0008449
RFWD2	12:55.4-56Mb	0.12622845	0.0002363	TP53	16:1.4-1.9Mb	0.11306321	0.000596	COL27A1	1:117.9-119.9Mb	0.10817287	0.0008469
MFRP	1:76.7-78.7Mb	0.1259672	0.0002407	F13A1	1:117.9-119.9Mb	0.11305433	0.0005963	RB1	19:48.9-49.3Mb	0.10816537	0.0008473
PADI2	1:76.7-78.7Mb	0.12584223	0.0002428	RB1	18:10.6-12.1Mb	0.11288442	0.0006041	PCDHA1	6:105.1-107.8Mb	0.10810167	0.0008509
MMP2	17:49.8-50.5Mb	0.12537706	0.0002505	THBS1	22:21-21.6Mb	0.11287811	0.0006045	SLC8A3	11:77.1-78.6Mb	0.10807334	0.0008526
CDH6	17:31.8-32.5Mb	0.12513766	0.0002555	RB1	18:1.5-3.5Mb	0.11284327	0.000606	ZNF615	1:76.7-78.7Mb	0.10806724	0.000853
DLEC1	1:121.6-121.6Mb	0.12465155	0.0002643	PHF20L1	1:206.1-207.3Mb	0.11280367	0.0006079	CCDC18	1:121.6-121.6Mb	0.10792043	0.0008623
ITGAV	1:40.7-42.6Mb	0.12451389	0.000267	RB1	19:56.1-56.8Mb	0.11268348	0.0006131	CEP250	7:63.7-64.4Mb	0.10787663	0.0008652
SLCO1A2	5:100.7-107.2Mb	0.12413367	0.0002742	RRP12	1:39.7-40.7Mb	0.112674	0.0006136	MMP2	17:48.3-48.9Mb	0.10787458	0.0008653
ICE1	22:22.6-22.8Mb	0.12335998	0.0002896	TRIM71	12:10.6-11.5Mb	0.11260085	0.0006167	CYTH3	17:15.4-16.1Mb	0.10786819	0.0008657
PAN2	12:25.1-27Mb	0.12330622	0.0002907	PLEKHA4	1:76.7-78.7Mb	0.11255958	0.0006184	SLC24A1	12:55.4-56Mb	0.10784817	0.0008669
CNTN4	1:119.9-121.6Mb	0.12312997	0.0002943	FAM153A	5:114.5-116.6Mb	0.11255296	0.0006187	MIB1	13:48.3-50Mb	0.1077734	0.0008718
COMP	6:105.1-107.8Mb	0.12311022	0.0002948	TP53	16:2.2-2.6Mb	0.11246384	0.0006228	ZNF534	11:99.1-102.8Mb	0.10774831	0.0008733
DNAH2	1:121.6-121.6Mb	0.12304151	0.0002959	HCRTR2	5:100.7-107.2Mb	0.11246227	0.000623	HECTD4	1:55.2-58.6Mb	0.10772605	0.0008747
ETAA1	2:191-195.6Mb	0.12288525	0.0002991	NCOA6	11:55.7-56.2Mb	0.11229781	0.0006304	TSEN2	1:121.6-121.6Mb	0.10769685	0.0008766
CFLAR	1:119.9-121.6Mb	0.12254458	0.0003062	ZNF224	12:10.6-11.5Mb	0.11227445	0.0006314	FAM171B	12:55.4-56Mb	0.10769127	0.000877
ANGPT1	11:59.4-60.4Mb	0.12240059	0.0003089	GPRIN3	12:65.8-68.2Mb	0.11224498	0.000633	PMM1	4:3.9-6.6Mb	0.10769118	0.000877
RPS6	1:73.3-76.6Mb	0.12235289	0.0003098	ABCC11	5:100.7-107.2Mb	0.11217975	0.0006359	FAM171A1	6:11.5-14.6Mb	0.10768778	0.0008771
LTBP1	1:40.7-42.6Mb	0.1221672	0.0003139	RRP12	1:206.1-207.3Mb	0.11207187	0.0006409	DSCAML1	19:7.1-7.9Mb	0.10762825	0.000881
ABCC11	5:114.5-116.6Mb	0.12179447	0.0003229	C1QL4	1:121.6-121.6Mb	0.11202944	0.0006432	NLN	2:191-195.6Mb	0.10758902	0.0008835
MITF	17:37-38.3Mb	0.12178992	0.000323	T	1:76.7-78.7Mb	0.11200548	0.0006441	ZMIZ1	19:27.6-29.5Mb	0.10758895	0.0008835
MDC1	1:121.6-121.6Mb	0.12167358	0.0003257	PWP2	1:121.6-121.6Mb	0.11198886	0.0006449	KIFAP3	4:143.6-146.1Mb	0.10754642	0.0008862
ZFYVE9	1:40.7-42.6Mb	0.1214636	0.00033	PRUNE2	11:55.7-56.2Mb	0.11196948	0.0006457	ATP2B1	1:206.1-207.3Mb	0.10754625	0.0008862
TRIM58	12:10.6-11.5Mb	0.12141185	0.0003311	HEATR5A	1:121.6-121.6Mb	0.11184995	0.0006512	ANKRD35	1:121.6-121.6Mb	0.10752557	0.0008875
MAOA	17:37-38.3Mb	0.12136937	0.0003323	ATIC	1:49.3-51.8Mb	0.11181118	0.0006533	TIAM1	1:121.6-121.6Mb	0.10751157	0.0008884
ZNF341	11:55.7-56.2Mb	0.12136403	0.0003324	IFT172	7:63.7-64.4Mb	0.11180214	0.0006536	TTC7A	22:22.6-22.8Mb	0.10750061	0.0008893
PADI1	5:114.5-116.6Mb	0.12133753	0.000333	POU6F2	6:6.7-10.1Mb	0.1117115	0.000658	HNRNPDL	1:121.6-121.6Mb	0.10745723	0.0008921
ELAVL4	11:77.1-78.6Mb	0.12098568	0.0003412	ITGB4	1:170.1-171.8Mb	0.11165664	0.0006605	MSI2	1:55.2-58.6Mb	0.1074532	0.0008923
TTC21B	1:76.7-78.7Mb	0.12071211	0.0003482	SLC38A2	22:22.8-23.4Mb	0.11164951	0.0006607	PTPN22	7:112.3-116.3Mb	0.10743734	0.0008933
ZKSCAN1	11:55.7-56.2Mb	0.12042834	0.0003549	HUS1	12:10.6-11.5Mb	0.11162755	0.0006617	ABHD16A	11:105.2-108.6Mb	0.10741826	0.0008946
NPTX1	7:112.3-116.3Mb	0.12015139	0.0003617	LDLR	1:119.9-121.6Mb	0.11158237	0.0006642	RSC1A1	5:114.5-116.6Mb	0.10734811	0.0008993
ZFYVE9	1:39.7-40.7Mb	0.11972112	0.0003731	ROCK2	2:92-92Mb	0.11158068	0.0006643	PSMD1	1:76.7-78.7Mb	0.10734202	0.0008996
TMEM184A	12:10.6-11.5Mb	0.11970394	0.0003733	CTAGE1	11:55.7-56.2Mb	0.11156173	0.0006655	TP53	16:0-0.5Mb	0.10731069	0.0009014
OR8B12	12:41.7-43.8Mb	0.11965388	0.0003747	MMP2	17:39.1-40.1Mb	0.11155895	0.0006655	UBE4A	12:10.6-11.5Mb	0.10729702	0.0009023
SLC4A1	5:114.5-116.6Mb	0.11964719	0.0003749	COL2A1	13:48.3-50Mb	0.1115321	0.0006668	PAN2	12:27-29Mb	0.1072787	0.0009037
TMC4	12:10.6-11.5Mb	0.11962968	0.0003755	ATIC	1:53.9-55.2Mb	0.11150685	0.0006682	PAPD7	2:179.1-182.9Mb	0.10727659	0.0009039
PARP10	11:55.7-56.2Mb	0.11915363	0.0003885	SNX19	6:6.7-10.1Mb	0.11147788	0.0006697	CCDC191	1:53.9-55.2Mb	0.10722846	0.0009067
UGT1A5	1:76.7-78.7Mb	0.1190196	0.0003923	ARHGAP26	1:53.9-55.2Mb	0.11147263	0.0006699	KDM3B	7:112.3-116.3Mb	0.10722484	0.0009069
STAG3	1:53.9-55.2Mb	0.11897187	0.0003937	STAG3	1:52.8-53.9Mb	0.11145335	0.0006707	CEMIP	6:105.1-107.8Mb	0.107219	0.0009072
LTN1	16:35.6-35.9Mb	0.11896116	0.000394	DHX35	1:117.9-119.9Mb	0.1114165	0.0006726	OR5H15	7:38.7-42.8Mb	0.107171	0.0009102
ARMC2	12:10.6-11.5Mb	0.1188766	0.0003964	SPECC1	12:10.6-11.5Mb	0.1113797	0.0006741	MMP8	12:65.8-68.2Mb	0.10712518	0.0009133
TRPA1	19:27.6-29.5Mb	0.1187958	0.0003986	PLD5	17:38.4-39.1Mb	0.11136776	0.0006746	FOXP1	12:10.6-11.5Mb	0.1070969	0.0009149
IGSF9B	22:22.6-22.8Mb	0.11875429	0.0003997	KDM1A	22:22.6-22.8Mb	0.11133148	0.0006765	ZNF394	1:117.9-119.9Mb	0.10707081	0.0009164
FRMD6	17:48.3-48.9Mb	0.11866996	0.0004021	TANC2	17:39.1-40.1Mb	0.11118222	0.0006837	IGSF9B	22:21-21.6Mb	0.10704698	0.000918
GMPS	17:31.8-32.5Mb	0.11864673	0.0004028	HIST1H2BC	11:55.7-56.2Mb	0.11116415	0.0006844	ARID1B	1:47-49.2Mb	0.1070238	0.0009198
ITGA2B	12:10.6-11.5Mb	0.11862315	0.0004034	THOC5	1:76.7-78.7Mb	0.11107994	0.0006887	ALMS1	4:184.3-185.6Mb	0.10696547	0.000924
RABEP2	1:121.6-121.6Mb	0.11861664	0.0004037	MYO18A	12:9.7-10.6Mb	0.11103809	0.0006912	ABCC12	1:76.7-78.7Mb	0.10692654	0.0009268
MARVELD3	17:31.8-32.5Mb	0.1186121	0.0004038	BAZ1A	13:48.3-50Mb	0.11103425	0.0006913	DOT1L	4:3.9-6.6Mb	0.10689195	0.000929
RRP12	1:38-39.7Mb	0.11838307	0.0004102	MEGF8	19:27.6-29.5Mb	0.11100688	0.0006926	ZFHX3	11:77.1-78.6Mb	0.10686664	0.0009305
DOCK9	19:9.7-10.3Mb	0.11835324	0.0004111	XKR7	15:25.1-25.2Mb	0.1109705	0.0006942	SYTL3	5:114.5-116.6Mb	0.10685963	0.0009311
CREB3	11:55.7-56.2Mb	0.11817324	0.0004158	SHPRH	7:18.8-22.4Mb	0.11095072	0.0006953	JARID2	21:39.4-41.9Mb	0.10684843	0.0009318
LMOD2	1:76.7-78.7Mb	0.11813141	0.0004172	VIL1	1:73.3-76.6Mb	0.11090137	0.0006977	LRRC37A3	12:65.8-68.2Mb	0.1068337	0.0009328

Table 2: Top mutation–SCNA associations (P<0.001) identified by CMI after accounting for tumor purity and mutation burden, continued.

ZNF615	1:119.9-121.6Mb	0.1181188	0.0004177	RIC1	16:28.8-29.5Mb	0.11086421	0.0006994	MIER1	1:119.9-121.6Mb	0.10681035	0.0009344
MMP2	17:37-38.3Mb	0.11780425	0.0004274	ZCCHC6	1:121.6-121.6Mb	0.11085088	0.0007003	SGIP1	12:65.8-68.2Mb	0.10680403	0.0009349
SMC2	22:22.8-23.4Mb	0.11769361	0.0004308	RB1	18:3.5-6.4Mb	0.11078718	0.0007038	ABHD3	7:116.2-117.6Mb	0.10678309	0.0009361
ESRP1	2:191-195.6Mb	0.11768252	0.0004311	HCRTR2	5:114.5-116.6Mb	0.11074227	0.0007062	RB1	19:51-51.4Mb	0.10676439	0.0009376
SYT3	13:48.3-50Mb	0.1176071	0.0004334	SLCO1C1	1:119.9-121.6Mb	0.11060405	0.0007128	KIF21A	11:60.4-61.4Mb	0.10674028	0.0009392
RIPK4	4:10.5-15.1Mb	0.11746504	0.0004375	CREBRF	5:114.5-116.6Mb	0.11052054	0.0007167	PAPSS2	12:55.4-56Mb	0.10670737	0.000941
ALDOA	1:121.6-121.6Mb	0.11746174	0.0004376	RB1	16:19.1-20.6Mb	0.11047447	0.0007188	STIL	1:40.7-42.6Mb	0.10668871	0.0009424
MAGEC3	12:65.8-68.2Mb	0.117334	0.0004411	PLEKHN1	12:55.4-56Mb	0.11046375	0.0007195	LETMD1	1:76.7-78.7Mb	0.10663958	0.0009462
ZNF626	1:189.9-193.8Mb	0.11722109	0.0004448	ADAMTS10	12:57.9-62.4Mb	0.11045327	0.00072	MFHAS1	22:22.8-23.4Mb	0.10663862	0.0009462
ADCY10	16:35.6-35.9Mb	0.11721819	0.0004449	DPH2	1:121.6-121.6Mb	0.11038368	0.0007237	MRGPRX1	17:37-38.3Mb	0.10663603	0.0009464
PABPC4	1:76.7-78.7Mb	0.11713356	0.0004471	USP4	22:22.6-22.8Mb	0.1103649	0.0007246	PAX3	1:76.7-78.7Mb	0.10663539	0.0009464
DCLK1	12:9.7-10.6Mb	0.11711779	0.0004475	USP40	1:189.9-193.8Mb	0.1103384	0.0007262	CROCC	1:73.3-76.6Mb	0.1066243	0.0009473
TSHZ2	5:114.5-116.6Mb	0.11709676	0.0004481	CD1E	22:30.5-31.3Mb	0.11027996	0.0007295	PHF2	21:14.1-16.5Mb	0.10659834	0.0009493
ARAP3	13:48.3-50Mb	0.11692467	0.0004532	RB1	19:55.4-56.2Mb	0.11023236	0.0007324	ABCC12	1:73.3-76.6Mb	0.10658928	0.0009499
OSBP2	1:76.7-78.7Mb	0.11686721	0.0004552	CASC1	2:179.1-182.9Mb	0.11022182	0.000733	PTPRG	17:60.1-61.6Mb	0.10658513	0.0009501
LRIG3	17:37-38.3Mb	0.11677339	0.0004581	METAP1D	1:121.6-121.6Mb	0.11009462	0.0007396	MCM3AP	6:6.7-10.1Mb	0.10658511	0.0009501
C7orf43	21:14.1-16.5Mb	0.11675695	0.0004586	MRGPRF	12:55.4-56Mb	0.11003471	0.0007428	FBLN1	1:117.9-119.9Mb	0.10657889	0.0009505
NR2C2	4:3.9-6.6Mb	0.11675554	0.0004587	SCN3A	15:71.5-72.9Mb	0.10995122	0.0007469	ZNF608	2:201.1-201.9Mb	0.10655213	0.000952
GRM7	12:57.9-62.4Mb	0.11674697	0.000459	WIPF1	1:76.7-78.7Mb	0.10992281	0.0007481	FAM47B	21:16.6-19.3Mb	0.10654338	0.0009529
DOCK9	19:7.1-7.9Mb	0.11664893	0.0004623	KBTBD7	19:27.6-29.5Mb	0.10987934	0.0007503	COL5A2	17:19.2-19.7Mb	0.10650049	0.0009555
PZP	2:191-195.6Mb	0.11658743	0.0004643	TYK2	12:56-56.4Mb	0.10981349	0.0007539	BRD9	19:27.6-29.5Mb	0.10648957	0.0009562
HCRTR2	7:116.2-117.6Mb	0.11658163	0.0004644	CIART	1:76.7-78.7Mb	0.10978539	0.0007553	MYO15A	17:36-36.9Mb	0.10648802	0.0009563
MSI2	1:52.8-53.9Mb	0.11644815	0.0004689	IWS1	7:56.6-57.4Mb	0.10978075	0.0007556	CFLAR	1:121.6-121.6Mb	0.10644839	0.0009591
SFXN2	12:10.6-11.5Mb	0.1164115	0.0004703	ADTRP	12:10.6-11.5Mb	0.10977577	0.0007558	CFAP43	12:56-56.4Mb	0.10642497	0.000961
SMC2	22:22.6-22.8Mb	0.11634989	0.0004725	PADI1	5:116.6-120.3Mb	0.10972342	0.0007585	FKBP5	7:63.7-64.4Mb	0.10639017	0.0009636
RTTN	2:201.1-201.9Mb	0.11626381	0.0004754	VWCE	13:50-51.9Mb	0.10972303	0.0007585	KCNIP1	4:3.9-6.6Mb	0.10634476	0.0009672
CROCC	1:76.7-78.7Mb	0.11616555	0.000479	ATIC	1:51.7-52.8Mb	0.10967891	0.0007608	ADAM22	11:55.7-56.2Mb	0.10628299	0.000972
RUNX1T1	7:63.7-64.4Mb	0.11613275	0.00048	DOCK6	2:127.9-130.2Mb	0.10966631	0.0007614	LTBP1	1:206.1-207.3Mb	0.10625651	0.0009738
BACH2	1:121.6-121.6Mb	0.11580611	0.0004907	STAG3	1:51.7-52.8Mb	0.10965199	0.0007624	GRM8	12:54-54.3Mb	0.10620953	0.0009775
C2CD5	7:63.7-64.4Mb	0.11573534	0.0004929	CNOT10	12:9.7-10.6Mb	0.10963354	0.0007633	KIAA1551	7:65.5-66.6Mb	0.10615495	0.0009816
KCTD3	1:119.9-121.6Mb	0.11571375	0.0004937	CASK	13:48.3-50Mb	0.10962982	0.0007635	HSPA14	17:37-38.3Mb	0.10612307	0.0009837
ADAM22	11:54.6-55.7Mb	0.11570385	0.000494	ADGRG1	1:121.6-121.6Mb	0.10961113	0.0007645	FAM98C	17:31.8-32.5Mb	0.10610714	0.0009847
PAN2	1:206.1-207.3Mb	0.11565342	0.0004954	MSI2	1:40.7-42.6Mb	0.10955414	0.0007679	CAND2	17:58.1-59.2Mb	0.10610635	0.0009847
KIF21B	17:58.1-59.2Mb	0.11532752	0.0005074	PLCL2	6:105.1-107.8Mb	0.10952207	0.0007701	ECEL1	5:100.7-107.2Mb	0.10609104	0.0009857
BCL9L	11:55.7-56.2Mb	0.11529767	0.0005087	ZNF599	12:62.5-64.2Mb	0.10951234	0.0007706	MUC3A	1:119.9-121.6Mb	0.10608491	0.000986
EEF2K	3:85.8-90.3Mb	0.11522733	0.0005111	TP53	16:3.1-3.5Mb	0.10949728	0.0007714	SPICE1	1:40.7-42.6Mb	0.10605326	0.0009881
ICAM5	7:63.7-64.4Mb	0.11497293	0.0005205	MPRIP	1:121.6-121.6Mb	0.10949213	0.0007716	PADI2	1:73.3-76.6Mb	0.10597635	0.0009937
ACP4	1:117.9-119.9Mb	0.11494942	0.0005217	ITPRIPL1	11:54.6-55.7Mb	0.10946796	0.0007728	ANKRD30A	4:87.3-88.7Mb	0.10593333	0.0009965
ABCC3	1:40.7-42.6Mb	0.11486154	0.0005246	ADGRL1	7:63.7-64.4Mb	0.10943381	0.0007746	DNM1P47	1:119.9-121.6Mb	0.10591656	0.0009974
ZNF479	13:48.3-50Mb	0.11483498	0.0005257	ZC3H13	1:76.7-78.7Mb	0.10940956	0.0007758	TPCN1	1:117.9-119.9Mb	0.10590494	0.0009984
ANO8	16:11.3-12.6Mb	0.11475735	0.0005287	ARHGAP33	1:119.9-121.6Mb	0.10940654	0.0007759	CNOT10	12:10.6-11.5Mb	0.10590409	0.0009984
TP53	16:1.9-2.2Mb	0.11475688	0.0005288					ETAA1	2:179.1-182.9Mb	0.10589338	0.0009991

7 Evaluation of permutation schemes under simulated conditional-null

Evaluating conditional dependency also relies on whether the permutation scheme preserves the dependence structure between the test variables and condition variable(s). We therefore evaluated alternative permutation schemes for CMI under simulated conditional-null settings in which

$$X \perp Y \mid Z,$$

while both X and Y depend on Z. This addresses the situation in which an apparent marginal association between X and Y arises from their shared dependence on Z, even though no residual association remains after conditioning on Z.

We first considered two nonlinear conditional-null examples. In both examples, the stochastic components of X and Y were generated independently, so that X and Y were conditionally independent given Z, despite nonlinear dependence of both variables on Z.

```
# Example 1
# n represents the vector size
z  <- rnorm(n)
ex <- rnorm(n, sd = 0.5)
ey <- rnorm(n, sd = 0.5)

x <- z + z^2 + ex
y <- -z + z^2 + ey

# Example 2
# n represents the vector size
z  <- rnorm(n, mean = 0, sd = 1)
ex <- rnorm(n, mean = 0, sd = 0.5)
ey <- rnorm(n, mean = 1, sd = 0.5)

x <- z + ex
y <- z * ey
```

We compared two permutation strategies. The first was a global permutation of the tested variables while keeping the condition variable fixed. This corresponds to the permutation strategy used in the two use cases in the main text. Global permutation breaks the association between X and Y, and disrupts the original dependence between the permuted variables and Z.

The second strategy was a conditional permutation. Specifically, samples were first stratified into bins according to Z, and X was then permuted only within each Z-bin. We refer to this strategy as `permute-X-within-Z-bin`. This procedure is intended to preserve the local dependence between X and Z, and between Y and Z, while breaking the residual association between X and Y within strata of Z. It is the permutation structure required by a conditional-independence null. For each example, we evaluated the empirical distribution of P-values across different sample sizes. The number of bins and the B-spline order used for CMI estimation were fixed at $B = 8$ and $s = 2$, respectively.

In Example 1, `permute-X-within-Z-bin` produced approximately uniform P-values when $n = 100$ (Fig. 22). However, as the sample size increased, the P-value distribution shifted toward zero, indicating increased Type I error under this simulated conditional null. This behavior is consistent with the fact that conditioning by discrete bins does not guarantee Type I error control [15]. By contrast, the global permutation strategy was more conservative at smaller sample sizes and became closer to uniform as the sample size increased (Fig. 22).

In Example 2, `permute-X-within-Z-bin` strategy produced approximately uniform P-value distributions across the evaluated sample sizes (Fig. 23). Overall, these simulations suggest that the behavior of conditional permutation can depend strongly on the functional form of the X-Z and Y-Z relationships, whereas global permutation tends to be more conservative.

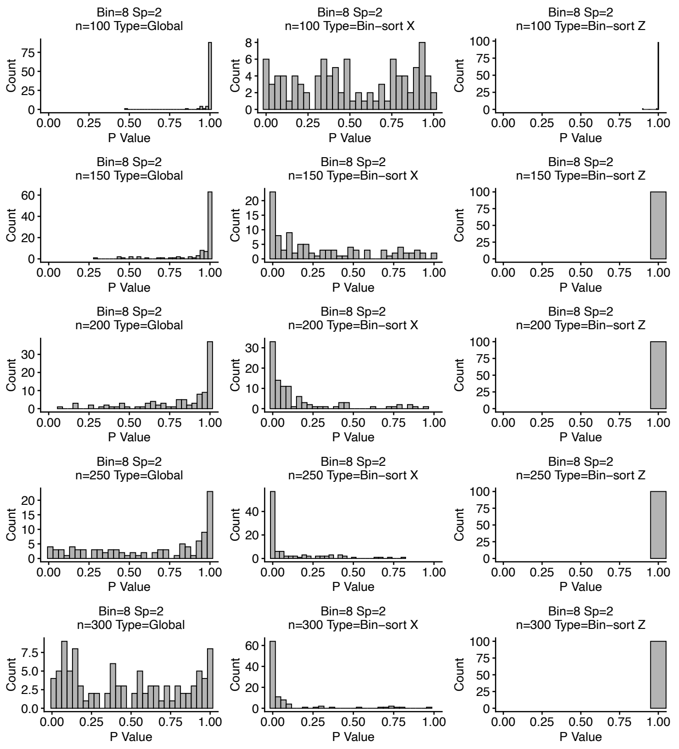

Figure 22: Empirical P-value distributions under the conditional-null model in Example 1. X and Y are conditionally independent given Z, while both variables depend nonlinearly on Z. Left: global permutation; middle: `permute-X-within-Z-bin`; right: `permute-Z-within-Z-bin`.

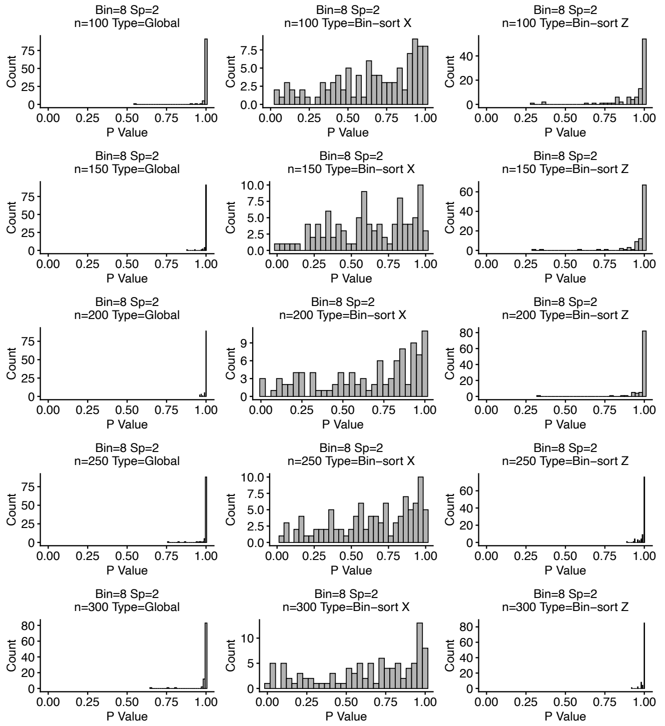

Figure 23: Empirical P-value distributions under the conditional-null model in Example 2. X and Y are conditionally independent given Z, while both variables depend on Z. Left: global permutation; middle: `permute-X-within-Z-bin`; right: `permute-Z-within-Z-bin`.

We next considered two additional examples in which X and Y retain a residual association after

conditioning on Z. In these non-null examples, the global permutation strategy remained more conservative than permute-X-within-Z-bin (Fig. 24 and Fig. 25). The permute-X-within-Z-bin strategy generally produced smaller P-values, reflecting higher sensitivity.

```
# Example 3
# n represents the vector size
z  <- rnorm(n)
k  <- rnorm(n)
ex <- rnorm(n, sd = 0.5)
ey <- rnorm(n, sd = 0.5)

x <- z + z^2 + k + ex
y <- -z + z^2 + k^2 + ey

# Example 4
# n represents the vector size
z  <- rnorm(n)
k  <- rnorm(n)
ex <- rnorm(n, sd = 0.5)
ey <- rnorm(n, sd = 0.5)

x <- z + k + ex
y <- z * k + ey
```

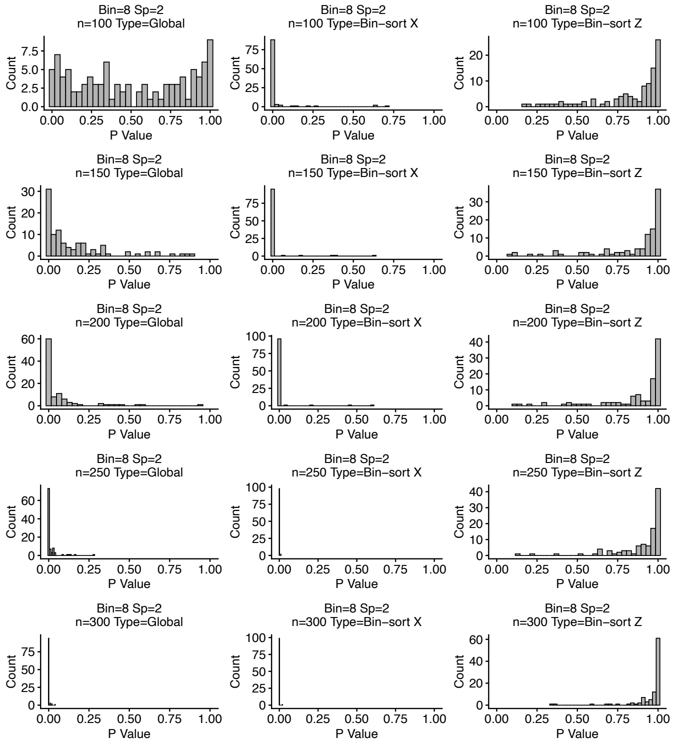

Figure 24: Empirical P-value distributions under the non-null model in Example 3, where X and Y remain associated after conditioning on Z. Left: global permutation; middle: permute-X-within-Z-bin; right: permute-Z-within-Z-bin.

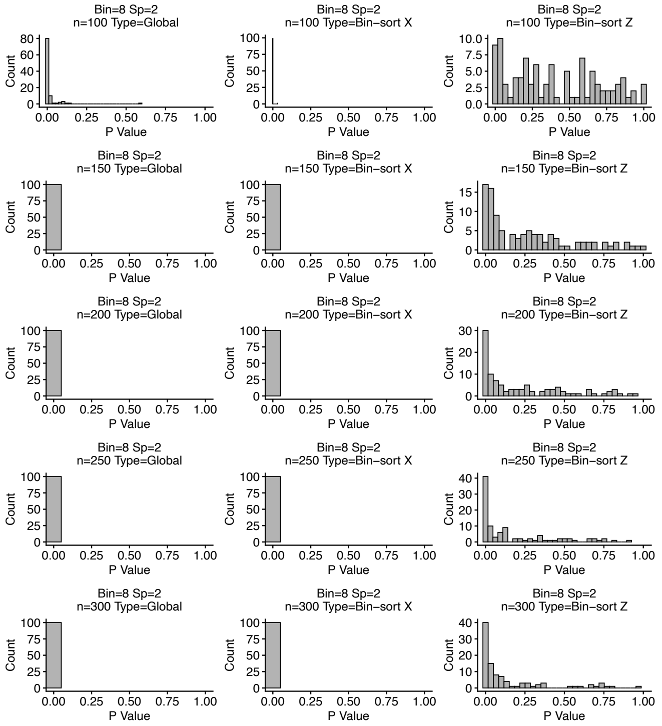

Figure 25: Empirical P-value distributions under the non-null model in Example 4, where X and Y remain associated after conditioning on Z. Left: global permutation; middle: `permute-X-within-Z-bin`; right: `permute-Z-within-Z-bin`.

We also evaluated a third strategy in which X and Y were kept fixed while Z was permuted within bins defined by Z (`permute-Z-within-Z-bin` strategy). This strategy was overly conservative in our simulations (Fig. 22, Fig. 23, Fig. 24 and Fig. 25). Even in Example 3, where X and Y were designed to be associated after conditioning on Z, the resulting P-values were centered at 1 (Fig. 24). We therefore do not consider this strategy appropriate for evaluating statistical significance for CMI.

Finally, we applied `permute-X-within-Z-bin` to use case 2 from the main text. Compared with the global permutation strategy, the within-Z permutation identified more statistically significant cell-type pairs across almost all combinations of bin number and spline order (Fig. 26 and Fig. 3). In particular, the association between Tregs and the other lymphocyte populations (CD4$^+$ cells, and CD8$^+$ T cells) became statistically significant in bin-spline order pair of (8,4), (12,2), (12,3), and (12,4). These results were consistent with the simulation results above as well as previous publication [15]: `permute-X-within-Z-bin` can increase sensitivity, but may also be less conservative and may not be able to control Type I error [15].

Taken together, these analyses show that the choice of permutation scheme can affect the statistical significance of CMI-based conditional association measurement. The results of the simulated examples suggested that global permutation was generally more conservative, whereas `permute-X-within-Z-bin` was more sensitive but could become loose under some nonlinear conditional-null models. Because real biological data, particularly cancer data, may contain diverse and complex nonlinear dependence structures, we do not treat either strategy as uniformly superior across all applications. Instead, `conMItion` provides both global permutation and conditional permutation as an user-defined option (with default setting of global permutation).

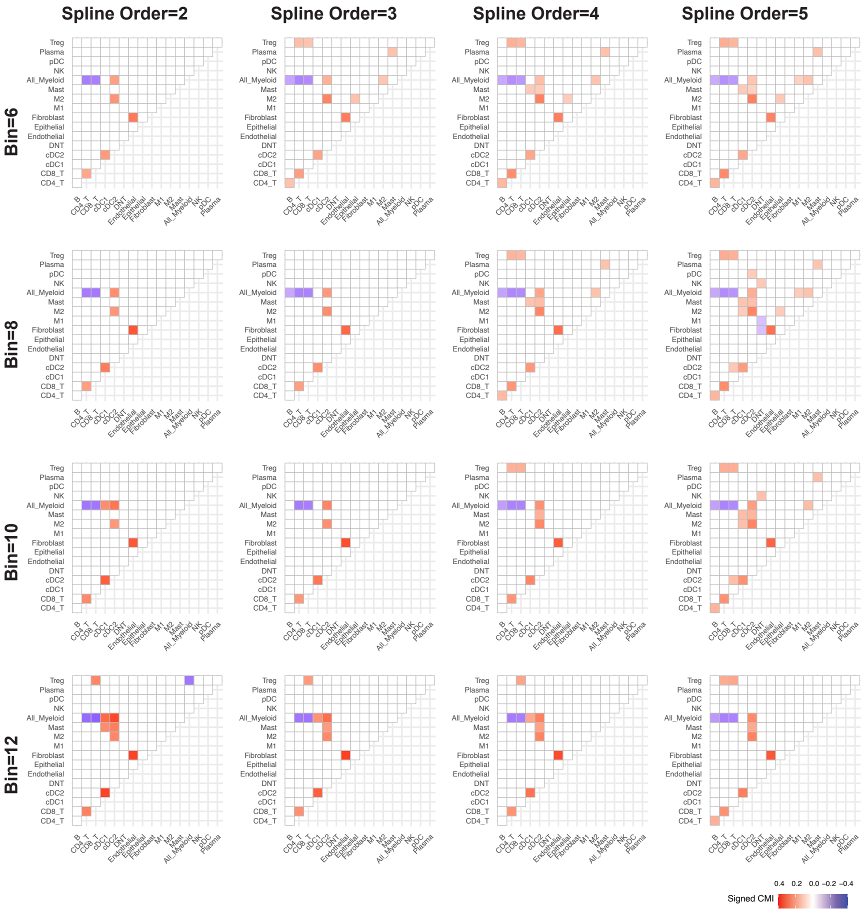

Figure 26: Sensitivity analysis of signed CMI estimates across bin numbers and spline orders using `permute-X-within-Z-bin`. Pairwise cell-type associations in the lung carcinoma cell-fraction dataset were evaluated using signed CMI under different parameter settings. Rows correspond to different bin numbers ($B = 6, 8, 10$, and 12), and columns correspond to different B-spline orders ($s = 2, 3, 4$, and 5). Each panel shows the upper-triangular matrix of signed CMI values between cell-type pairs. Red indicates positive associations, blue indicates negative associations, and color intensity reflects the magnitude of the signed CMI estimate. Only statistically significant associations are shown (FDR < 0.2).

References

[1] Daub, C.O. et al. Estimating mutual information using B-spline functions–an improved similarity measure for analysing gene expression data. *BMC Bioinformatics*, 5(1):118, 2004.

[2] Colaprico, A. et al. TCGAbiolinks: an R/Bioconductor package for integrative analysis of TCGA data. *Nucleic Acids Research*, 44(8):e71, 2016.

[3] Ellrott, K. et al. Scalable Open Science Approach for Mutation Calling of Tumor Exomes Using Multiple Genomic Pipelines. *Cell Systems*, 6(3):271–281.e7, 2018.

[4] Taylor, A.M. et al. Genomic and Functional Approaches to Understanding Cancer Aneuploidy. *Cancer Cell*, 33(4):676–689.e3, 2018.

[5] Carter, S. L. et al. Absolute quantification of somatic DNA alterations in human cancer. *Nature Biotechnology*, 30:413–421, 2012.

[6] Weizmann Institute of Science. Lung - 3CA. *Weizmann Institute of Science Website*, `https://www.weizmann.ac.il/sites/3CA/lung`, accessed on 2023-10-03.

[7] Hao, Y. et al. Dictionary learning for integrative, multimodal and scalable single-cell analysis. *Nature Biotechnology*, 2023.

[8] McElreath, R. *Statistical Rethinking–A Bayesian Course with Examples in R and STAN.* 2nd ed. CRC Press, Boca Raton, 2020.

[9] Kurt, Z. et al. Impacts of the different spline orders on the B-spline association estimator. In *13th IEEE International Conference on BioInformatics and BioEngineering*, pp. 1–6, 2013.

[10] Draper, N.R. and Smith, H. *Applied Regression Analysis.* 3rd ed. Wiley, New York, 1998.

[11] Cohen, J. et al. *Applied Multiple Regression/Correlation Analysis for the Behavioral Sciences.* 3rd ed. Lawrence Erlbaum Associates, Mahwah, NJ, 2003.

[12] Tufail, M. et al. Immune evasion in cancer: mechanisms and cutting-edge therapeutic approaches. *Signal Transduction and Targeted Therapy*, 10(1):227, 2025.

[13] Du, W. et al. Extracellular matrix remodeling in the tumor immunity. *Frontiers in Immunology*, 14, 2024.

[14] Sun, S. et al. Transcription factor E4F1 as a regulator of cell life and disease progression. *Science Advances*, 9(39):eadh1991, 2023.

[15] Berrett, T. B. et al. The conditional permutation test for independence while controlling for confounders. *Journal of the Royal Statistical Society: Series B*, 82(1):175–197, 2020.
